# Supplementary material for: The novel molecular mechanism of pulmonary fibrosis: insight into lipid metabolism from reanalysis of single-cell RNA-seq databases
Source: Lipids Health Dis. 2024 Apr 3;23:98. doi: 10.1186/s12944-024-02062-8 (PMC10988923; doi:10.1186/s12944-024-02062-8)
Supplement: Supplementary file 2 — Supplementary material 2. [file 12944_2024_2062_MOESM2_ESM.pdf]

The_novel_molecular_mechanism_of_pulmonary_fibrosis__insight_into_lipid_metabolism_from_reanalysis_of_single_cell_RNA_seq_databases_

WORD COUNT	13869	TIME SUBMITTED	24-FEB-2024 07:10PM
		PAPER ID	107115711

The novel molecular mechanism of pulmonary fibrosis: insight into lipid metabolism from reanalysis of single-cell RNA-seq databases

Shi *et al*.

Abstract

Pulmonary fibrosis (PF) is a severe pulmonary disease with limited available therapeutic choices. Recent evidence increasingly points to abnormal lipid metabolism as a critical factor in PF pathogenesis. Our latest research identifies the dysregulation of low-density lipoprotein (LDL) is a new risk factor for PF, contributing to alveolar epithelial and endothelial cell damage, and fibroblast activation. In this study, we first integrative summarize the published literature about lipid metabolite changes found in PF, including phospholipids, glycolipids, steroids, fatty acids, triglycerides, and lipoproteins. We then reanalyze two single-cell RNA-sequencing (scRNA-seq) datasets of PF, and the corresponding lipid metabolomic genes responsible for these lipids' biosynthesis, catabolism, transport, and modification processes are uncovered. Intriguingly, we found that macrophage is the most active cell type in lipid metabolism, with almost all lipid metabolic genes being altered in macrophages of PF. In type 2 alveolar epithelial cells, lipid metabolic differentially expressed genes (DEGs) are primarily associated with the cytidine diphosphate diacylglycerol pathway, cholesterol metabolism, and triglyceride synthesis. Endothelial cells are partly responsible for sphingomyelin, phosphatidylcholine, and phosphatidylethanolamines reprogramming as their metabolic genes are dysregulated in PF. Fibroblasts may contribute to abnormal cholesterol, phosphatidylcholine, and phosphatidylethanolamine metabolism in PF.

Therefore, the reprogrammed lipid profiles in PF may be attributed to the aberrant expression of lipid metabolic genes in different cell types. Taken together, these insights underscore the potential of targeting lipid metabolism in developing innovative therapeutic strategies, potentially leading to extended overall survival in individuals affected by PF.

Keywords: Pulmonary fibrosis, lipid metabolism, lipid metabolomic gene，single-cell RNA-sequencing reanalysis

1. Introduction

Pulmonary fibrosis (PF) is irreversible, with high mortality and few effective treatments [1]. The scarring causes the lung tissues to become thick and stiff, making it harder to absorb oxygen into the bloodstream. Clinically, dyspnoea, dry cough, fatigue and exhaustion are the main manifestations of patients with PF. Medical imaging studies suggest that PF lungs show high levels of collagen fiber deposition, severe alveolar loss and destroyed lung architecture.

The incidence of PF is approximately 3-18 per 100,000 people in idiopathic PF (IPF) and 3-24 per 100,000 people in autoimmune PF [2, 3]. Notably, global incidents of silicosis, a subtype of PF, have risen by 64.6%, from 84,821 cases in 1990 to 138,965 in 2019 [4]. PF encompasses various etiological subtypes, including IPF of unknown cause, connective tissue disease-related PF (notably systemic sclerosis and dermatomyositis patients, occupational PF (e.g., silica-related silicosis) due to environmental exposure, virus-induced PF (exemplified by SARS-CoV-2), and genetic spontaneous PF. Genetic spontaneous PF can be further classified into familial IPF, typically associated with surfactant proteins A and C (SP-A and SP-C) mutations, sporadic IPF linked to mutations in poly(A)-specific ribonuclease (PARN), telomerase reverse transcriptase (TERT), regulator of telomere elongation helicase 1 (RTEL1), telomerase RNA component (TERC), and gain-of-function mucin 5B (MUC5B)-

induced IPF. Mechanistically, familial IPF mutations such as SP-C induce endoplasmic reticulum (ER) stress, and an acquired impairment in macroautophagy-dependent proteostasis and mitophagy increases alveolar epithelial type II (AT2) cell susceptibility to injury [5]. The sporadic mutations cause cells to suffer telomere DNA damage, while the MUC5B mutation induces distal airway epithelial distension [6-8]. Through decades of efforts investigating the etiology of the development of PF, complex underlying mechanisms of pathophysiological PF have been revealed, including cell senescence, alveolar epithelial injury, endothelial barrier disturbance, chronic inflammation, and activation of macrophages and fibroblasts [8-11]. Currently, the pharmacologic treatment options for PF are limited, with only the FDA-approved pirfenidone and nintedanib available [12]. However, both two drugs are highly toxic to the liver and kidneys. It requires a systemic-level approach to uncover detailed molecular and cellular alterations in PF toward understanding its pathology. Fortunately, recent advancements in scRNA-seq technology have enabled a more precise cell type-specific transcriptional analysis, offering promising opportunities for integrating and interpreting changes across cell types and genes, thereby aiding in the discovery of novel therapeutic targets.

Currently, increasing evidence suggests that lipid metabolism disorders are involved in PF, including abnormalities in low-density lipoprotein (LDL) metabolism [13], sphingosine-1-phosphate (S1P), and so on [14-18]. Epidemiological studies have

indicated that patients with pneumoconiosis, often a result of environmental pollution, exhibit a distinctive serum metabolite profile. Metabolites such as phosphatidylethanolamine (22:6/18:1) and N-tetradecanoylsphingosine have been proposed as potential biomarkers, while 1,2-dioctanoylthiophosphatidylcholine, phosphatidylcholine (18:1/20:1) and indole-3-acetamide have been identified as potential indicators for the staging of pneumoconiosis [19]. Additionally, diseases with significant lipid metabolism abnormalities, like cardiovascular disease and obesity, have been notably linked with PF [20, 21]. For instance, lipid risk factors common in cardiovascular disease are also prevalent in IPF. Saturated fatty acid diets have been associated with an elevated prevalence of PF, and obesity doubles the risk of developing this condition. However, the specific roles and molecular mechanisms of lipid metabolic reprogramming in PF remain inadequately understood. Therefore, the investigation in PF lipidomics of detail PF-related cells is beneficial for understanding the underlying mechanisms of PF in-depth and providing clues for new therapeutic strategies.

Here, we first integratively summarized recent findings on dysregulated lipid metabolites in PF and then focused on the molecular regulation of these lipid metabolites by re-analysing two single-cell RNA-sequencing datasets of PF. We also investigate the importance of lipid and related metabolic mechanisms in PF pathophysiology. Our study reveals that lipid metabolism dysregulation is present in

various PF-related cell types and mediates abnormal cellular functions. Furthermore, impaired lipid metabolism is closely correlated with clinical manifestations such as disease severity and survival. In addition, different cell types have an apparent predilection for lipid dysregulation, for example, phospholipid dysregulation is more likely to occur in AT2 cells. These insights suggest that understanding lipid metabolism dysregulation patterns at various disease stages and across different cell types could be beneficial for PF interventions.

2. Physiopathologic mechanisms underlying PF

Fibroblasts, known for their role in excessive extracellular matrix (ECM) deposition during wound healing, are pivotal effector cells in fibrosis [22]. In response to internal and external signals, the morphological stretches of fibroblasts are increased [3]. These cells secrete ECM components, primarily collagen, exacerbating fibrosis [23]. Indeed, the process of PF is far more than that simple, and merely controlling fibroblast collagen metabolism cannot effectively inhibit PF.

The damage of endothelium, alveolar epithelium, and alveolar macrophage activation are essential for the initiation of PF [3]. During the early stage of PF, the injury of pulmonary capillary endothelial cells (ECs) initiates the apoptosis process

and thus destroys the endothelial barrier. Meanwhile, the living apoptosis-resistant endothelial cells are activated and express many inflammatory cytokines and chemokines, contributing to inflammatory cell recruitment and infiltration. The activated ECs can also secrete profibrotic factors, especially transforming growth factor beta 1 (TGF-β1) to induce fibroblast-like changes and activate perivascular fibroblasts. In the late stage of EC injury, the fibroblast repair processes are dominant. The abnormal repair processes collectively result in aberrant angiogenesis, vasculogenesis, tissue hypoxia, and fibrosis [24].

The alveoli, composed mainly of alveolar epithelial type I (AT1) and AT2 cells, characterized by their delicate and thin structure. Like ECs, some epithelial cells undergo apoptosis in response to injurious stimuli, while others become activated. The apoptotic loss of AT1 and AT2 cells results in the reduction of alveoli, thereby impairing pulmonary function. Though AT1 differentiates from AT2 to remedy the alveoli regeneration, this process is broken by persistent and chronic inflammation in the condition of PF. Instead, the physiological repair of AT2-AT1 differentiation is superseded by fibroblast repair. Finally, the injury loci are full of ECM accompanied by the alveoli disappearance. Besides apoptosis, the AT1 and AT2 also undergo the activation program by responding to the interleukins such as monocyte chemoattractant protein-1 (encoded by CCL-2), interleukin-8 (encoded by CXCL8), interleukin-6 (IL6), and TGF-β1, leading to inflammation, accumulation of fibroblast

-like AT1 and AT2 cells and fibroblast activation [8].

Pulmonary macrophages include alveolar macrophages (AMs) and interstitial macrophages (IMs) [25]. When the alveolar epithelium is injured, macrophages are activated to become activated macrophages and alternatively activated macrophages (AAMs), which consist of four subtypes, including AAM2a, AAM2b, AAM2c, and AAM2d [26, 27]. Macrophages are highly heterogeneous and complex to classify. The traditional categories are M1, M2, or AM, AAM. Recently scRNA-seq analyses have divided lung macrophages into inflammatory macrophages, airspace macrophages, and profibrotic macrophages or other specific gene high-expressing macrophages, including secreted CCL2, inhibin subunit beta A (INHBA), fatty acid binding protein 4 (FABP4), phosphoprotein 1 (SPP1), serpin family G member 1 (SERPING1), interleukin 1 receptor type 2 (IL1R2), interleukin 1 beta (IL1B), and ficolin 1 (FCN1) macrophages [25, 28-32]. Both activated macrophages and AAMs have been reported to mediate PF by releasing pro-inflammatory and profibrotic factors to activate continuous fibroblasts and promote myofibroblast proliferation [33].

Senescent cells undergo cell cycle arrest and phenotypic changes. However, they are metabolically active and contribute to many diseases. The contribution of cellular senescence to PF, and in particular its acceleration in this condition, is being increasingly recognized. The hallmarks of senescence, including telomere shortening,

genomic instability, mitochondrial dysfunction, impaired autophagy, defective nutrient sensing, and epigenetic alterations, are significantly enriched in PF. Cell senescence is an important phenomenon affecting endothelial cells, AT2 cells, fibroblasts, and macrophages. Senescent cells drive the senescence-associated secretory phenotype (SASP) to affect surrounding cells. This secretion leads to the activation of proinflammatory and profibrotic pathways, ultimately contributing to PF.

Taken together, PF involves abnormalities in the endothelium, alveolar epithelium, fibroblasts, and macrophages (**Figure 1**).

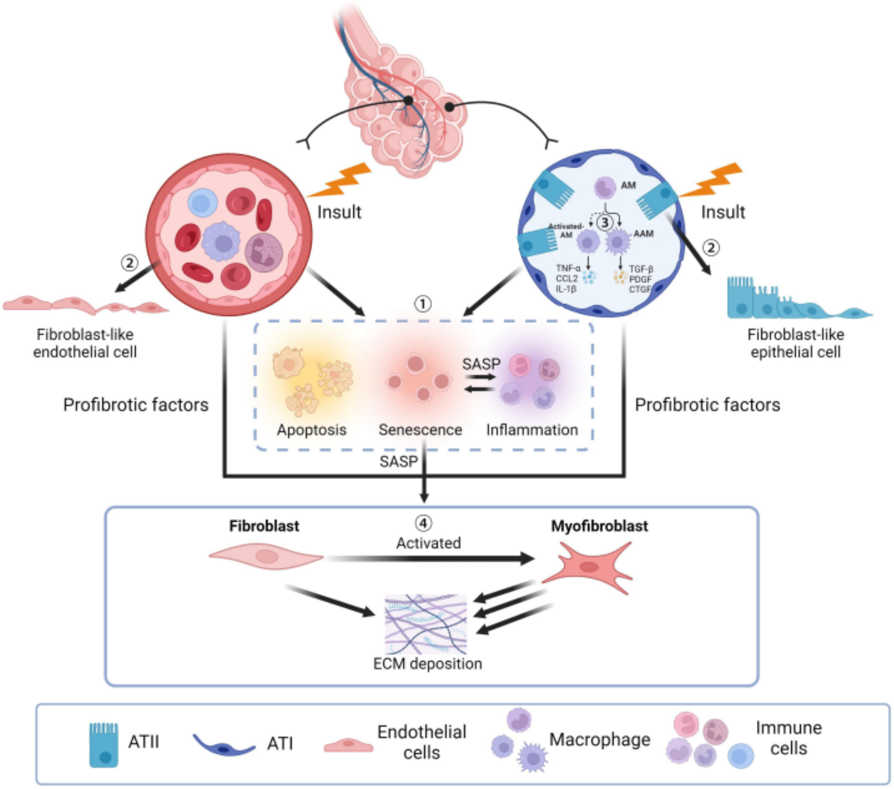

Figure 1. The mechanism of PF. ① Excessive apoptosis of endothelial and epithelial cells leads to lung injury and releases various pro-inflammatory and profibrotic factors. Injured EC and alveolar epithelial cells also undergo senescence, inducing an SASP phenotype, which further enhances the pro-inflammatory and profibrotic effects. ② The apoptosis-resistant endothelial and epithelial cells undergo an activated process to obtain fibroblast-like properties. ③ The polarisation of macrophages confers AM and IM cells to differentiate into activated macrophages, which produce abundant pro-inflammatory and profibrotic factors and further promote PF. ④ Over-proliferation and hyperactivation of fibroblast lead to ECM deposition and fibrosis formation.

3. Impacts of lipid metabolism during PF

3.1 Phospholipids

Phospholipids are a predominant component of pulmonary surfactants, comprising about 10% specific surfactant proteins and 90% lipids. Pulmonary surfactants primarily function to reduce surface tension within the alveoli. Phosphatidylcholine (PC), with dipalmitoylphosphatidylcholine (DPPC) comprising half of it, makes up 70-75% of surfactant phospholipids and provides surface-active properties. These surfactant phospholipid components are critical to the functionality and stability of the alveoli and are reprogrammed in PF [34]. A recent plasma lipidomics study has shown that almost all the surfactant phospholipid species are reduced in IPF in comparison to controls, including PC, phosphatidylserine (PS), phosphatidylethanolamines (PE), phosphatidylinositol (PI), and phosphatidylglycerol (PG) [34, 35]. The decline of surfactant phospholipids has also been validated in lung biopsy samples from irradiation-induced PF patients [36]. Interestingly, Shabarinath Nambiar *et al.* observed higher plasma PC levels in progressive IPF than in stable cases, potentially linked to severe epithelial cell damage in advanced IPF [37]. These divergent PC patterns may be intimately associated with disease progression and could provide essential insights for diagnosis and treatment.

Phospholipids play an important role in fibrogenesis. Luis G. Vazquez-de-Lara *et al*.

recently reported that PE could inhibit collagen deposition by promoting apoptosis, inducing a dose-dependent Ca^{2+} signaling and mitigating bleomycin (BLM)-induced PF in mice [38]. In this study, PE treatment was started 1 day after BLM injection in mice and continued 6 times, and the degree of fibrosis was assessed on day 21. It was found that PE mainly decreased collagen expression in fibroblasts. Since fibroblast activation is a late effect of PF, we suggest that the remission effect of PE on PF may be therapeutic rather than preventive. Another study reported by Stefanie Preuß *et al* showed that PG could prevent fibrosis by inhibiting alveolar epithelial injury and fibrosis responses by reducing secretory phospholipase A2 [39]. PG was administered ex vivo to the lungs of 2-6 days old domestic piglets. The results showed that PG could not only inhibit alveolar damage but also inhibit the TGF-β1 secretion and other fibrotic factors, suggesting a potential role in PF prevention and treatment.

Conversely, lysophospholipids have a profibrotic effect by inducing apoptosis of alveolar cells, vascular permeability, migration and activation of fibroblasts [40]. Surfactant phospholipids are beneficial for lung hemostasis. However, the modified phospholipids are pathogenetic and mainly removed by alveolar macrophages (AMs) in PF, especially the oxidized phospholipids (ox-PLs). Increasing evidence indicates that ox-PLs actively contribute to the commencement and advancement of PF. Freddy Romero *et al.* showed that ox-PLs accumulate in AMs of human patients and mouse models of PF and induce an M2 phenotype transition of AMs, secreting high levels of

TGF-β1, ultimately exacerbating BLM-induced PF [41]. Moreover, recent studies further showed that ox-PLs could induce ferroptosis and thus promote PF [42-44]. The uptake of ox-PLs is mediated by CD36 molecule (CD36). Amounts of studies demonstrated that CD36 promoted ER stress, cell death in AT2 cells, and PF [45, 46].

By reanalyzing scRNA-seq datasets (GSE136831 and GSE135893) of endothelial, AT2, fibroblast and macrophage cells, we found that the reduced surfactant lipids are partly attributed to the downregulated surfactant lipid metabolism-related genes in PF. Among those downregulated genes, CHK (choline kinase, α/β), choline phosphotransferase 1 (CHPT1), phosphate cytidylyltransferase 1A, choline (PCYT1A), and phosphatidylethanolamine N-methyltransferase (PEMT) are involved in the PC biosynthetic process through the Kennedy pathway (**Figure 2**). Besides, the generation of PE is also mediated by the Kennedy pathway, and the critical genes are ethanolamine kinase (ETNK1/2), PCYT2 (phosphate cytidylyltransferase 2), and choline/ethanolamine phosphotransferase 1 (CEPT1). In contrast, 8 lecithin-cholesterol acyltransferase (LCAT) and phospholipase are responsible for the decomposition of PC and PE. The scRNA-seq datasets show that a series of surfactant genes are dysregulated in AT2 cells and macrophages of PF (**Table 1**). In addition, the expressions of CDP-diacylglycerol synthase (CDS1/2), phosphatidylglycerol phosphate synthase 1 (PGS1), phosphatidylserine synthase 1 (PTDSS1/2), CDP-diacylglycerol-inositol 3-phosphatidyl transferase (CDIPT), which are responsible for

the synthesis of PS, PI, and PG by CDP-DAG pathway (**Figure 2**), are altered as well (**Table 2**). Besides the *de no* synthesis, phospholipids can also be converted from their corresponding lysophospholipids, which are mediated by the lysophosphatidylcholine acyltransferases family (LPCATs). In turn, phospholipids can be converted to lysophospholipids in the presence of phospholipase A2s (PLA2s).

Although lung fibroblasts, macrophages, and ECs are not primary sources of surfactant phospholipids, alterations in phospholipid gene expression in these cells merit attention. For instance, it was found that AT2 cells can uptake cholesterol from extracellular LDL via the LDL receptor (LDLR) [13]. Pulmonary lipofibroblasts are characterized by their lipid droplets and are located in the alveolar interstitium. They contain cortical contractile filaments and are related to contractile interstitial cells and are beneficial for alveolar development [47]. These lipofibroblasts can transport lipids to AT2 cells via the parathyroid hormone-related protein (PTHRP) signaling pathway, which is activated by stretch-sensitive AT2 cells and directs the differentiation of mesenchymal and alveolar epithelial cells [48, 49]. In addition to these signaling pathways, other cells can also deliver phosphatidylcholine to epithelial cells in the form of exosomes [50]. These lipids likely contribute to the synthesis of AT2 surfactant lipids, underscoring the importance of considering the phospholipid synthesis capacity of other cells. Taken together, all of these results suggested that surfactant phospholipids were reduced in PF and thus exacerbated the PF course.

Targeting the surfactant phospholipids metabolism by mediating the activity of Kennedy and cytidine diphosphate-diacylglycerol (CDP-DAG) pathways is a promising strategy for PF.

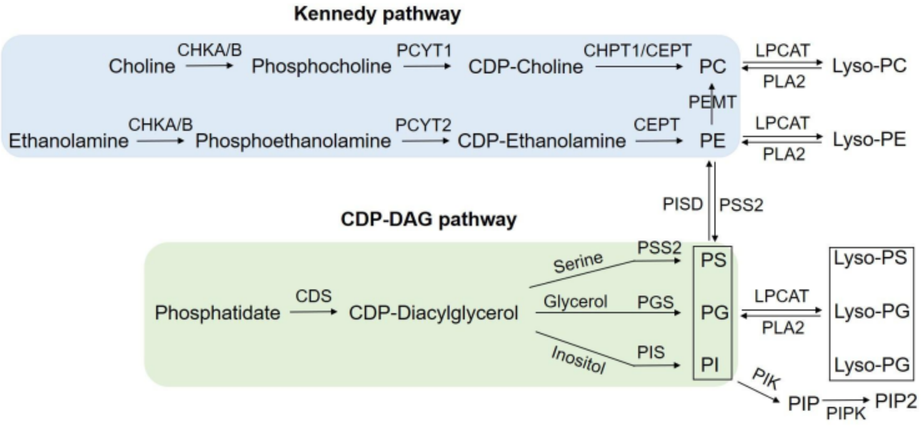

Figure 2. The metabolites and genes involved in the Kennedy pathway and CDP-DAG pathway. The synthesis of PC and PE via the Kennedy pathway, and the synthesis of PS, PG and PI through the CDP-DAG pathway.

Table 1. DEGs of Kennedy pathway in PF cells.

Cell Type	AT2		Fibroblast		Macrophage		Endothelium	
Significance	Fold Change	*P* value	Fold Change	*P* value	Fold Change	*P* value	Fold Change	*P* value
CHKA	/	/	0.57 (FB)	2.67 E-02	0.06 (IM)	3.97 E-05	/	/
					-0.15 (AM)	9.64 E-10		
CHKB	/	/	/	/	-0.79 (AM)	2.73 E-09	/	/
PCYT1A	/	/	/	/	-0.20 (AM)	5.84 E-18	/	/
CHPT1	-0.95	4.80 E-84	-0.41 (FB)	1.15 E-02	0.17 (IM)	2.52 E-08	-1.58	1.09 E-58
			-1.90 (MyoF)	8.82 E-55	-0.27 (AM)	8.89 E-13		
ETNK2	/	/	/	/	-0.90 (AM)	1.71 E-02	/	/
PCYT2	-0.78	1.04 E-45	-1.13 (FB)	1.45 E-02	/	/	-0.58	6.21 E-04
CEPT1	0.47	1.51 E-03	/	/	-0.16 (IM)	6.90 E-09	-0.55	1.92 E-05
					-0.77 (AM)	2.55 E-140		
PEMT	-0.24	2.68 E-03	/	/	0.17 (IM)	5.00 E-05	0.29	4.97 E-03
					0.24 (AM)	2.34 E-04		
LCAT	1.36	5.96 E-19	/	/	-0.39 (AM)	4.38 E-02	-0.69	6.71 E-03
PLA2G4C	/	/	/	/	-0.67 (AM)	3.36 E-22	/	/
PLA2G16	-0.44	4.91 E-54	/	/	-0.27 (IM)	3.26 E-35	-0.28	1.42 E-09
					-0.18 (AM)	3.29 E-51		

Abbreviation. **FB**: Fibroblasts; **MyoF**: Myofibroblasts; **IM**: interstitial macrophages; **AM**: alveolar macrophages.

Table 2. DEGs of CDP-DAG pathway in PF cells.

Cell Type	AT2		Fibroblast		Macrophage		Endothelium	
Significance	Fold Change	*P* value	Fold Change	*P* value	Fold Change	*P* value	Fold Change	*P* value
SELENOI	/	/	/	/	0.09 (IM)	4.28 E-02	/	/
CDS1	-0.22	6.50 E-05	/	/	-0.28 (AM)	2.08 E-03	/	/
CDS2	/	/	/	/	-0.24 (AM)	6.78 E-26	/	/
PTDSS1	-0.34	1.54 E-03	/	/	-0.21 (AM)	4.76 E-15	/	/
PTDSS2	/	/	/	/	-0.34 (IM)	1.59 E-12	/	/
					-0.39 (AM)	5.77 E-07		
PISD	0.43	2.36 E-03	-0.84 (MyoF)	2.80 E-02	-0.14 (IM)	7.56 E-06	/	/
					-0.45 (AM)	1.35 E-37		
PGS1	-0.28	3.27 E-05	/	/	-0.33 (IM)	1.16 E-121	-0.31	4.47 E-04
					-0.43 (AM)	1.62 E-48		
CDIPT	/	/	/	/	0.19 (IM)	2.35 E-10	/	/
					0.18 (AM)	1.93 E-04		

Abbreviation. **FB**: Fibroblasts; **MyoF**: Myofibroblasts; **IM**: interstitial macrophages; **AM**: alveolar macrophages.

Sphingomyelin (SM) is synthesized via SM synthases (SMSs, SGMS1). In the

presence of sphingomyelinases (SMases), which are encoded by sphingomyelin phosphodiesterases (SMPD1-4) genes, SM is hydrolyzed to ceramide (Cer). Previous studies have demonstrated Cer-mediated cell infection, inflammation, and death susceptibility in cystic fibrosis [51]. N-acyl sphingosine amidohydrolases (ASAH1/2) facilitate the degradation of ceramide into sphingosine, which could be phosphorylated by sphingosine kinase 1 (SPHK1) to generate S1P. The actions of S1P are predominantly mediated by S1P receptors (S1PRs), including S1PR1, S1PR2, and S1PR3 [52]. SM is the most abundant sphingolipid and has particularly high levels in the brain [53]. Indeed, moderate levels of SM were also found in the lung [54], and the metabolic pathway was disrupted in PF. Decreased sphingolipid metabolites in IPF have been reported by Yidan D Zhao [55]. In addition, a series of sphingolipid metabolism-related genes are reduced in IPF lungs, including SMPD1, SMPD4, SPHK1, S1PR1, S1PR4, and S1P lyase (SGPL1) [55]. A report by Long Shuang Huang *et al.* showed that S1P lyase (S1PL, encoded by SGPL1), an enzyme that catalyzes S1P to phosphoethanolamine, is negatively correlated with PF severity but positively correlated with survival rate. Moreover, overexpression of S1PL reduces S1P levels, enhances fibroblast autophagy, attenuates lung fibroblast activation, and effectively inhibits BLM-induced PF [16]. Researches have also shown that inactivation of the SPHK1/S1P/S1PR signaling attenuates mouse PF by reducing ECM deposition in fibroblasts [14, 17, 56-58]. However, Rachel S. Knipe *et al.*

recently found that endothelial-specific *S1pr1* deletion suppresses sphingosine-1-phosphate metabolism and shows increased peripheral lymphocyte numbers by increasing vascular permeability and exacerbating BLM-induced PF [59]. The distinct roles of S1P in different PF-related cell types may explain this discrepancy. We then analyzed the scRNA-seq datasets and found that the changing trends of these SM metabolism-related genes in different cell types were inconsistent (**Table 3**). Nevertheless, it gives us a hint and suggests that the function of S1P metabolism could be considered in a manner that is dependent on the cell type, and results obtained from whole lung tissues should be carefully interpreted. In summary, these results underscore the important role of SM metabolism in PF, with the processes involved depicted in **Figure 3**.

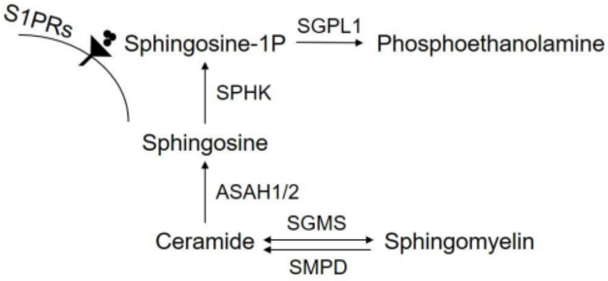

Figure 3. The metabolites and genes involved in SM metabolism. ASAHs catalyze ceramide to sphingosine, which is phosphorylated by SPHK to produce S1P. S1P can bind to S1PRs on the cell surface to regulate cell function or be degraded by SGPL1.

Table 3. DEGs of SM metabolism pathway in PF cells.

Cell Type	AT2		Fibroblast		Macrophage		Endothelium	
Significance	Fold Change	*P* value	Fold Change	*P* value	Fold Change	*P* value	Fold Change	*P* value
SGMS1	/	/	/	/	-0.18 (IM)	8.54 E-46	/	/
					0.06 (AM)	1.66 E-03		
SGMS2	0.49	1.06 E-24	/	/	0.40 (IM)	2.25 E-176	/	/
					0.29 (AM)	7.56 E-117		
SMPD1	/	/	/	/	-0.29 (AM)	2.54 E-09	-0.49	7.33 E-08
SMPD4	/	/	/	/	-0.24 (IM)	1.87 E-13	-0.59	3.98 E-06
					-0.37 (AM)	1.23 E-17		
ASAH1	-0.37	6.67 E-121	/	/	-0.05 (IM)	4.47 E-13	-0.36	1.86 E-13
					-0.02 (AM)	1.62 E-02		
ASAH2	/	/	/	/	-0.81 (AM)	1.82 E-04	/	/
SPHK1	/	/	/	/	-0.94 (IM)	2.30 E-301	0.23	2.84 E-02
					-0.89 (AM)	7.96 E-12		
SPHK2	0.54	1.18 E-04	/	/	/	/	/	/
S1PR1	/	/	/	/	0.82 (AM)	3.93 E-02	-0.31	1.32 E-30
S1PR3	/	/	/	/	-0.72 (IM)	4.05 E-08	/	/

S1PR4	1.00	3.73 E-04	/	/	/	/	-0.66	3.74 E-05
SGPL1	0.74	6.80 E-08	/	/	-0.50 (IM)	3.67 E-291	/	/
					-0.11 (AM)	2.75 E-03		

3.2 Glycolipids

Miguel Arias-Guillen *et al.* discovered that glycosphingolipids mediate profibrotic TGF-β/SMAD signaling in human lung fibroblasts. Suppression of glycosphingolipid synthesis was found to decrease ECM deposition and myofibroblast transformation. Similarly, Toru Kimura *et al.* demonstrated that a glycolipid derived from marine sponges, α-galactosylceramide, attenuates BLM-induced PF. This attenuation occurs through the regulating of several cytokines, including TGF-β, interferon-gamma (IFN-γ), connective tissue growth factor (CTGF), and macrophage inflammatory protein-2 released by natural killer T cells [60]. The metabolism and the roles of glycolipids in PF remain largely unknown.

3.3 Steroids

Steroids include sterols (e.g., cholesterol), bile acids, steroid hormones (e.g. adrenal corticosteroids, androgens, estrogens), etc.

3.3.1 Cholesterol metabolism

(1) Alterations of cholesterol and its derivatives in PF

As early as 1996, E Fireman *et al*. reported that cholesterol was deposited in the bronchoalveolar lavage fluid (BALF) of IPF patients [61]. Besides, increased cholesterol is observed in BLM-induced PF [41]. In addition to cholesterol levels, Tomohiro Ichikawa *et al*. reported that one cholesterols' derivative, 25-hydroxycholesterol, could promote myofibroblast differentiation and ECM deposition via a TGF-β/nuclear factor kappa B (NF-κB) dependent manner [62]. Feng Yan *et al.* observed lower plasma levels of the sterol lipid 20:1-Glc-Sitosterol in IPF patients than in healthy donors. Moreover, three sterol lipids (16:1 stigmasterol ester, 3-hydroxyvitamin D3, and 20:1-glc-sitosterol) have been identified as correlating with IPF [35]. These results demonstrate that abnormal cholesterol metabolism is a risk factor in the pathogenesis of PF.

(2) Abnormal regulation of cholesterol metabolism

The sterol regulatory element-binding protein 2 (SREBP2) tightly regulates *de novo* cholesterol synthesis. In sterol-deficient cells, SREBP2 increases cholesterol synthesis by generating oxysterol ligands for LXRα/β (encoded by NR1H3 and NR1H2, respectively) [63]. Interestingly, although excessive cholesterol has been observed to be deposited in the BALF of PF, the expressions of cholesterol-synthesis-related genes decrease in PF lungs, including hydroxymethyl-glutaryl coenzyme A reductase

(HMGCR) and SREBPs [41]. Moreover, overexpression of SREBP2 could suppress lung fibroblast proliferation, ER stress, and attenuates PF [64, 65]. Among the target genes of SREBP2, many of them can regulate non-steroid lipid metabolism, and are antifibrotic in PF, such as LDLR, fatty acid synthase (FASN), SCD, etc [66]. In contrast, another study reported that SREBP2 is markedly increased in IPF lung specimens. Endothelial-specific transgenic of SREBP2 activated the TGF-β and Wnt signaling and fibrotic genes such as smooth muscle (α-SMA), vimentin, snail family transcriptional repressor 1 (Snai1), neural cadherin, and actin alpha 2. This led to EC overgrowth, ECM deposition, stress fiber formation, and exacerbated BLM-induced PF [24].

The uptake of extracellular cholesterol is mediated by LDLR. LDLR governs the uptake of cholesterol packaged with apolipoprotein B (ApoB), especially LDL-C particles, from the blood [67-69]. In lung tissue, specifically on AT2 cells, LDLR takes up peripheral LDL particles for surfactant synthesis, a process that is impaired in acute and chronic lung injury [67, 68, 70]. Mice lacking LDLR (*Ldlr-/-*) exhibit impaired lung development compared to wild-type (WT) mice [71]. Consistent with our and other previous studies, a disrupted LDL-LDLR metabolic axis was found in PF patients [13, 72]. Further *in vivo* and *in vitro* studies of these aberrations revealed their contributions and mechanisms in PF. We develop a combined treatment with a statin and an anti-proprotein convertase subtilisin/kexin type 9 (PCSK9) antibody that

significantly reduces the severity of PF, more effectively than either treatment alone, by increasing LDLR and lowering LDL in mice.

For cholesterol homeostasis, lung cells either expel excess cholesterol or store it as cholesteryl esters in lipid droplets. ATP-binding cassette subfamily A member 1 (ABCA1) is expressed widely throughout the body, with the lung having the second highest expression after the liver [73]. ABCA1 in macrophages facilitates the removal of cholesterol and prevents excessive cholesterol deposition in the lungs [74]. Lipid-free circular apolipoprotein A-I (apoA-I) receives cholesterol effluxed via ABCA1 and forms the nascent high-density lipoprotein (HDL) particles on the cell membrane. Nascent HDL matures after acquiring cellular cholesterol effluxed through ATP-binding cassette subfamily G member 1 (ABCG1) and ABCA1 [75]. In the liver, circular HDL binds to hepatic scavenger receptor class B type I (SR-BI) and is cleared, whereas cholesteryl ester transfer protein (CETP)-mediated cholesterol transfer from HDL to LDL is cleared by hepatic LDLR. This process of cellular cholesterol disposal is termed reverse cholesterol transport (RCT) [67]. To prevent intracellular free cholesterol accumulation, acetyl-CoA acetyltransferase 1 (ACAT)-mediated cholesterol esterification directs cholesterol toward storage [76]. Esterification is also necessary to balance free cholesterol and cholesteryl esters.

Alessandro Venosa *et al.* found that reduced ABCA1 and ABCG1 in macrophages

from nitrogen mustard-induced PF mice [77]. Disabled cholesterol efflux and esterification were found in the BLM-induced PF model. Moreover, deleting the lipid efflux transporter ABCG1 could reduce pulmonary lipid clearance and worsen lung fibrosis [41]. Besides, our previous study revealed that plasma HDL levels are both decreased in PF patients and mice, further indicating dysfunctional cholesterol efflux in PF. In addition, HDL particles are negatively correlated with the death of IPF [78]. scRNA-seq dataset of PF further shows these cholesterol effluxes and esterification genes are decreased in macrophages and AT2 cells compared to healthy individuals (**Table 4**). The exact role of ACATs in PF remains to be determined, although increased ACATs have been associated with atherosclerosis [79].

The current discourse on cholesterol homeostasis has received considerable attention owing to its crucial role in an expanding spectrum of diseases, extending beyond traditional cardiovascular disorders to include pulmonary diseases [13], various cancers [80], and Alzheimer's disease [81]. Intriguingly, our research has shown that in addition to cardiovascular disease, cholesterol reduction may be a viable therapeutic approach for PF. Nevertheless, numerous critical inquiries regarding cholesterol metabolism in PF persist, particularly concerning the underlying mechanisms. The question of whether cholesterol levels are regulated in a manner akin to that in the lungs remains unresolved. Unraveling this uncertainty is imperative for a full understanding of the relationship between cholesterol and PF.

Table 4. DEGs of cholesterol metabolism in PF cells.

Cell Type	AT2		Fibroblast		Macrophage		Endothelium	
Significance	Fold Change	*P* value	Fold Change	*P* value	Fold Change	*P* value	Fold Change	*P* value
SREBF1	/	/	-0.57 (FB)	4.15 E-03	-0.20 (AM)	1.66 E-03	-0.58	1.91 E-06
SREBF2	-0.14	1.27E -02	/	/	0.23 (IM)	3.51 E-42	/	/
					0.07 (AM)	4.19 E-02		
NR1H2	-0.38	5.61 E-24	/	/	-0.13 (IM)	7.63 E-10	-0.24	5.95 E-04
					-0.30 (AM)	8.00 E-37		
NR1H3	/	/	/	/	0.55 (IM)	2.55 E-144	/	/
					-0.20 (AM)	1.08 E-14		
LDLR	-0.43	3.18 E-44	/	/	0.21 (IM)	1.14 E-18	/	/
					0.56 (AM)	9.63 E-59		
FASN	-0.59	5.43 E-130	/	/	/	/	/	/
SCD	/	/	-1.04 (FB)	7.92 E-03	0.32 (IM)	3.42 E-61	/	/
					-0.14 (AM)	2.80 E-21		
PCSK9	-1.03	5.02 E-44	/	/	/	/	/	/
ABCA1	/	/	-1.11 (FB)	3.04 E-33	-0.63 (IM)	0.00 E+00	/	/
			-0.54 (MyoFB)	5.29 E-03	-0.66 (AM)	5.32 E-233		
ABCG1	/	/	/	/	-0.04 (IM)	3.56 E-03	-0.25	6.74 E-03

					0.11 (AM)	5.00 E-28		
ACAT1	-0.52	7.51 E-55	/	/	0.07 (IM)	1.66 E-02	-0.17	1.44 E-02
					-0.17 (AM)	7.01 E-17		
ACAT2	-1.15	1.79 E-130	/	/	/	/	/	/

3.3.2 Bile Acid Metabolism in PF

Bile acids (BAs) are synthesized by cytochrome P450 family 7 subfamily A member 1 (CYP7A1) and subfamily B member 1 (CYP8B1). BAs serve as crucial mediators of inflammation and fibrosis, exerting their effects via the activation of both nuclear and membrane G protein-coupled receptors [82]. Activation of farnesoid X receptor (FXR, encoded by the *NR1H4* gene) occurs upon its interaction with BAs or their derivatives. Notably, Obeticholic acid (OCA), a BA-derived agonist of FXR, is clinically advanced in its ability to suppress BA production in hepatocytes and enhance bile acid transport from hepatocytes, thus reducing hepatic exposure to BAs [83].

BAs have been detected in cystic fibrosis lungs [84], with their levels being closely associated with lung function parameters [85]. Yidan D. Zhao *et al.* found elevated bile acid metabolites in pulmonary arterial hypertension (PAH) patients, suggesting that pulmonary vascular endothelial cells of CYP7B1 protein may partly drive the *de*

novo bile acid synthesis process [86]. Importantly, BAs could increase intracellular reactive oxygen species (ROS) production and subsequently induce EMT of AT2 and lung fibroblast activation *in vitro* through TGF-β/Smad3 signaling-dependent manners [87]. Consistently, microaspiration of BAs induces lung fibrosis through activating VEGF, CTGF, bFGF, and TGF-β pathways in rats [88]. Though BAs are profibrotic, FXR mediates inhibitory effects of inflammation and fibrosis in FXR-expressing organs [89, 90]. Indeed, FXR is confirmed to be expressed in AT2 cells [91]. *In vivo* treatment with OCA has been shown to effectively ameliorate BLM-induced pulmonary function loss and reverse lung fibrosis by attenuating EMT, reducing IL-6 and IL-1β, and downregulating profibrotic SNAI1 and TGF-β1 expression [92], even superior to those obtained with pirfenidone [93], highlighting FXR as a novel PF therapeutic target. The mechanisms of the opposite effect of BAs and FXR on PF need to be further investigated. In addition to FXR, the roles of other receptors of BA, like pregnane X receptor (PXR), Takeda G protein-coupled receptor (TGR5), and sphingosine-1-phosphate receptor 2 (S1PR2) in the lungs, remain unexplored. Collectively, these findings suggest that BA receptor agonists may be promising for alleviating inflammation- and fibrosis-related diseases.

3.3.3 Steroid hormones

Steroid hormones are broadly divided into two categories: corticosteroids and sex steroids, which are generally synthesised in the adrenal glands and gonads or placenta, respectively. These categories include glucocorticoids, mineralocorticoids, estrogens, androgens, and progestins-five types based on the receptors they bind.

The lungs can respond to hormones through steroid hormone receptors expressed in the lungs. The classical estrogen receptors include estrogen receptor alpha (ERα) and estrogen receptor beta (ERβ). Studies have indicated a high expression of ERβ in both alveolar and bronchiolar epithelial cells. Interestingly, both female and male ERβ knockout (*ERβ−/−*) mice exhibited decreased caveolin-1, while increased metalloproteinases, and TIMP metallopeptidase inhibitor 2 (TIMP2), and manifested defective alveogenesis, reduced lung volume, unexpanded alveoli, systemic hypoxia, and spontaneous fibrosis [94]. More recent work by Sharon Elliot has shown elevated pulmonary ERα levels in PF patients and mice. Mice harboring inactivated estrogen receptors develop BLM-induced lung fibrosis [95]. Additionally, progesterone receptor (PR) is positively stained in myofibroblasts in the scarred areas of IPF, implying PR could be a potential target in PF [96]. Steroid hormones can transfer to the lung through a circular system and thus act on lung fibrosis. For instance, plasma dehydroepiandrosterone (DHEA) is reduced in IPF patients and has been shown to

significantly inhibit PF characteristics. DHEA decreases fibroblast proliferation and increases apoptosis, likely through the intrinsic pathway involving caspase-9 activation. It also significantly inhibits fibroblast-to-myofibroblast differentiation, collagen production and fibroblast migration. [97]. Conversely, male sex hormones, or androgens, appear to exacerbate lung fibrosis following BLM administration [98]. Testosterone and 5α-dihydrotestosterone (DHT) are decreased significantly in the IPF group [99], but their roles in PF remain unclear.

Vitamin D (VitD) functions as a steroid hormone with inhibitory effects on inflammation and fibrosis, largely by modulating TGF-β, MAPK, and NF-κB pathways [100, 101]. IPF and other types of interstitial lung disease (ILD) patients display decreased serum VitD concentrations and lung Vitamin D receptor (VDR). VitD was also positively correlated with the diffusion capacity of the lungs for carbon monoxide (DLCO)% and predicted forced vital capacity (FVC)%, and negatively correlated with mortality of IPF [102].

In 2011, international guidelines for IPF recommended glucocorticoids in acute exacerbation IPF patients [103]. *In vivo* and *in vitro* experiments consistently demonstrate glucocorticoids enter lung cells through glucocorticoid receptors (GRα and GRβ, two isoforms encoded by nuclear receptor subfamily 3 group C member 1, *NR3C1*), and then suppress PF by blocking fibroblast TGF-β production [104, 105].

However, the contents of glucocorticoid receptors in IPF patients are lower than those in normal volunteers. What's worse is that IPF patients with lower glucocorticoid receptor levels are resistant to glucocorticoid treatment [106, 107]. In the realm of vascular diseases, such as cardiovascular disease and PAH, the mineralocorticoid receptor (MR) has been identified as a contributory factor. As a result, MR antagonism is considered a promising therapeutic approach [108, 109]. However, it has been observed that MR antagonism does not significantly alter the outcomes of COVID-19-related PF treatment [110]. Presently, there is a dearth of data regarding the potential involvement of mineralocorticoid hormones and their receptors in IPF and other forms of PF. DEGs of bile acid and steroid hormones metabolism-related genes were listed in **Table 5**.

In summary, steroid hormones and their receptors are involved in PF and may prove to be effective therapeutic targets in PF.

Table 5. DEGs of bile acid and steroid hormones metabolism in PF cells.

Cell Type	AT2		Fibroblast		Macrophage		Endothelium	
Significance	Fold Change	*P* value	Fold Change	*P* value	Fold Change	*P* value	Fold Change	*P* value
NR1H4	/	/	/	/	-0.72 (IM)	7.53 E-10	/	/

					-0.77 (AM)	9.47 E-04		
ESR2	/	/	/	/	-0.52 (IM)	3.54 E-10	3.32	3.83 E-07
					-0.44 (AM)	6.70 E-03		
ESR1	/	/	/	/	0.14 (IM)	2.67 E-04	/	/
					-0.58 (AM)	6.78 E-86		
VDR	/	/	/	/	-0.23 (IM)	5.89 E-32	/	/
					-0.50 (AM)	9.39 E-38		

3.4 Triglyceride (TG)

Lipofibroblasts (LFs) received more and more attention as they widely participate in various lung disorders, including PF [111]. Cell fate tracing experiments revealed that LFs originate from fibroblasts, and will transdifferentiate to myofibroblasts when exposed to a stimulus [112]. TG may act as a pivotal modulator of lung fibroblast homeostasis by contributing to the assembly of lipofibroblasts, which are characterised by lipid droplets (LDs) that undergo continuous cycles of synthesis and degradation. We found that diacylglycerol O-acyltransferase 1 (DGAT1), an essential enzyme responsible for the last step of TG synthesis, is specifically enriched in perilipin 2 (PLIN2)-positive LFs, rather than fibroblast, myofibroblast, or any other types of lung cells [112]. This suggests that TG is crucial for LF maintenance by

regulating LD synthesis and degradation. The schematic diagram of lipid droplet metabolism lipid droplet metabolism was shown in **Figure 4**, and the DEGs in PF responsible for LD metabolism are listed in **Table 6**. Furthermore, LDs function as a multifaceted organelle involved in various physiological and pathological processes, including ER stress, insulin resistance, autophagy, mitochondrial and nuclear function regulation, inflammatory response, and viral infection [113].

Studies have shown that lipid droplets can maintain the high activation of the lipogenic pathway of lipofibroblasts and convert myofibroblasts into fibroblasts with weak collagen-producing ability. [114]. Moreover, lipid droplets store large amounts of lipids, and lipid droplet-rich lipofibroblasts are physically adjacent to the alveoli and play a crucial role in alveolarisation [115]. Therefore, LDs may be significant in the resolution of PF and potentially in alveolar regeneration.

Taken together, the investigation focuses on the regulatory network and the roles of TG and LD metabolism in the lung may provide a new therapeutic approach for PF.

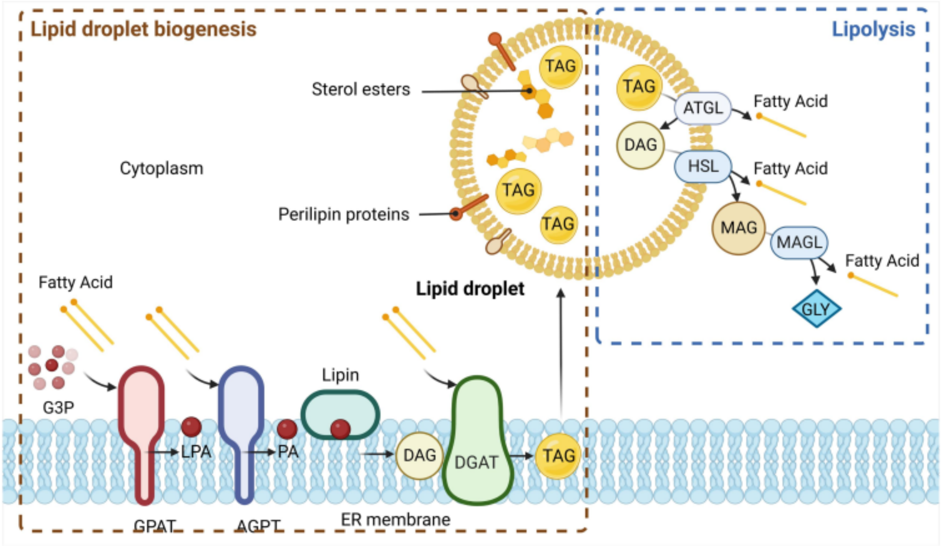

Figure 4 Regulation of Lipid Droplet Formation by TG. The left panel of this figure depicts the multi-step synthesis of triglycerides (TAGs). Initially, glycerol-3-phosphate acyltransferase (GPAT) catalyzes the biosynthesis of lysophosphatidic acid (LPA) with a preference for saturated fatty acids and glycerol-3-phosphate (G3P) as substrates. GPATs exist in two forms, the mitochondrial isoform (GPAT1/2) and the endoplasmic isoform (GPAT3/4). Next, 1-acylglycerol-3-phosphate O-acyltransferases (AGPATs) convert LPA to phosphatidic acid (PA). Following this, the enzyme lipin, a magnesium-ion-dependent phosphatidic acid phosphohydrolase, dephosphorylates PA to yield diacylglycerol (DAG). Diacylglycerol O-acyltransferases (DGAT1/2) catalyze DAG and fatty acyl-CoA to TAG. The biogenesis of lipid droplets commences with TG synthesis, which accumulates between the ER membrane's two leaflets. Proteins bound to the lipid droplet surface, such as perilipins (PLINs), localize to the phospholipid monolayer, while the neutral lipid core comprises triacylglycerols and sterol esters. The right panel illustrates the hydrolysis of TG. Adipose triglyceride lipase (ATGL), encoded by the PNPLA2 gene, initiates TAG degradation to produce DAG, which is subsequently hydrolyzed to monoacylglycerol (MAG) by

hormone-sensitive lipase (LIPE). LIPE also participates in steroid hormone synthesis by converting cholesteryl esters to free cholesterol. Finally, monoglyceride lipase (MGLL) hydrolyzes MAG to free fatty acids and glycerol.

Table 6. DEGs of lipid droplet metabolism in PF cells.

Cell Type	AT2		Fibroblast		Macrophage		Endothelium	
Significance	Fold Change	*P* value	Fold Change	*P* value	Fold Change	*P* value	Fold Change	*P* value
GPAT3	-0.72	3.40 E-04	/	/	-0.57 (IM)	1.01 E-06	-0.40	5.89 E-21
					-1.15 (AM)	3.20 E-03		
AGPAT4	0.79	2.46 E-02	/	/	0.31 (IM)	2.47 E-10	/	/
AGPAT5	/	/	/	/	/	/	0.80	5.41 E-09
AGPAT3	0.47	3.33 E-09	/	/	-0.35 (AM)	1.50 E-06	/	/
AGPAT1	0.73	2.91 E-12	/	/	/	/	/	/
AGPAT2	-0.35	8.81 E-49	/	/	/	/	/	/
PNPLA2	-0.25	9.30 E-09	/	/	-0.40 (IM)	1.92 E-03	/	/
					-0.35 (AM)	4.01 E-07		
LPIN2	/	/	/	/	-0.50 (AM)	3.34 E-31	/	/
LPIN3	1.29	8.21 E-16	/	/	/	/	/	/
MGLL	/	/	/	/	-0.46 (AM)	3.33 E-15	1.07	5.22 E-40

PLIN2	/	/	-0.37 (FB)	2.43 E-03	-0.40 (IM)	0.00 E+00	/	/
					-0.36 (AM)	0.00 E+00		
PLIN5	/	/	/	/	-0.49 (IM)	1.13 E-05	/	/
					-1.65 (AM)	3.63 E-02		
DGAT1	0.84	5.96 E-20	/	/	-0.34 (IM)	1.01 E-19	/	/
DGAT2	-1.10	2.05E -10	/	/	-1.32 (IM)	4.56 E-80	/	/
					-1.75 (AM)	4.05 E-73		

3.5 Fatty acids (FAs)

Recent studies have highlighted distinctive alterations in FA metabolism in PF, encompassing de novo synthesis, uptake, oxidation, and derivatization processes [116]. The initial step of *de novo* FA synthesis involves ATP citrate lyase (ACLY), which converts cytoplasmic citric acid into oxaloacetic acid and acetyl-CoA. Subsequently, acetyl-CoA carboxylase (ACC), a rate-limiting enzyme, allows acetyl-CoA to be carboxylated to malonyl-CoA. Finally, malonyl-CoA and acetyl-CoA are catalyzed by fatty acid synthase (FASN) to palmitic acid (PA). FAs can also be internalized via cell surface receptors, such as the CD36 receptor. Intracellular FAs bind to coenzyme A and are then shuttled to the mitochondria to start the FA oxidation (FAO) process, producing carbon dioxide and water in the presence of sufficient oxygen.

Yidan D Zhao *et al.* found increased free FAs but reduced carnitine shuttle, suggesting reduced mitochondrial β-oxidation in IPF. Hiroaki Sunaga *et al.* reported a significant downregulation of elongation of long-chain fatty acids family member 6 (Elovl6) in PF. Elovl6 knockdown altered the composition of oleic acid (OA), PA, and linoleic acid (LOA), resulting in heightened apoptosis, reactive oxygen species (ROS) production, and TGF-β1 secretion in AT2 cells, thereby exacerbating PF [117]. The pathogenic effect of PA on PF is also revealed by the Sarah G. Chu group [45]. In contrast, stearic acid markedly decreased p-Smad2/3 phosphorylation, ROS generation, and fibrosis [118]. The altered FA levels in IPF lung tissues have also been explored in this article and it found that PA, oleic acid, and LOA were elevated, while the level of stearic acid was significantly reduced compared to controls. PA, OA, or LOA significantly enhance the TGF-β1 induced fibrosis, whereas stearic acid significantly reduces it. Studies have demonstrated that the FA synthetic activator nuclear receptor subfamily (LXR)/SREBP-1c axis is linked to fibrosis [64, 65, 119]. A role for altered FA metabolism through the activation of FASN via the rapamycin-sensitive TGFβ1/mTORC1 pathway [120]. As a downstream of the LXR/SREBP-1c axis, FASN is downregulated in AT2 cells. AT2 cell-specific loss of FASN impaired mitochondria biogenesis and promoted PF [23]. However, FASN is required for TGF-β-induced profibrotic responses, and its inhibition not only mitigates fibrosis but also improves lung function. Stearoyl-CoA desaturase (SCD) desaturates saturated FA to

prevent lipotoxicity, ER stress and apoptosis caused by saturated FA [121]. Therefore, it is a theoretical benefit for PF via SCD manipulation. The schematic diagram of the metabolism of fatty acid is shown in **Figure 5**.

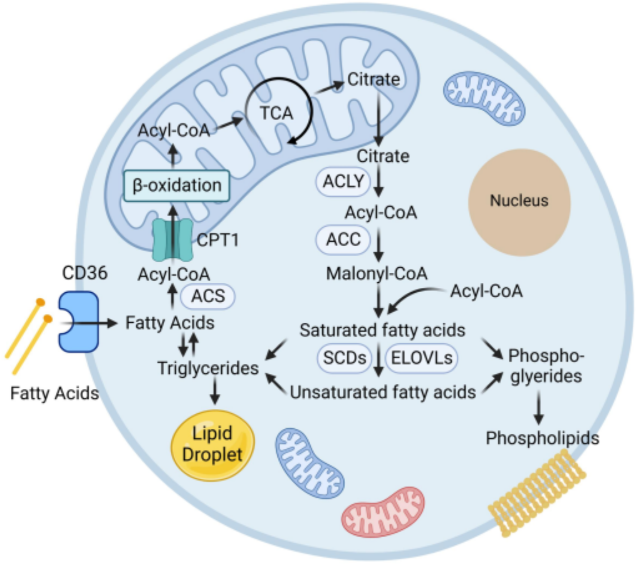

Figure 5 Regulation of FA Metabolism Pathways. ACLY utilizes cytoplasmic citrate to produce cytosolic acetyl-CoA, which is then used as a substrate by ACC to produce malonyl-CoA. FASN could convert acetyl-CoA and malonyl-CoA into long-chain saturated fatty acids for palmitate synthesis. SCD plays a critical role in the synthesis of unsaturated FAs, particularly oleic acid. The ELOVLs are involved in fatty acid elongation. In FA catabolism, acyl-CoA synthetases (ACSLs) first degrade long-chain FAs (LCFAs) to fatty acyl-CoA esters. Subsequently, mitochondrial carnitine palmitoyltransferases (CPT1/II), in conjunction with a carnitine-acylcarnitine translocase, induce the oxidation of LCFAs, which are ultimately broken down to acetyl-CoA via the β-oxidation pathway in the mitochondria.

3.6 Lipoproteins

Lipids can be stored in lipoproteins and transported to peripheral tissues. Altered lipoprotein levels have been reported to be associated with PF. Our and other groups consistently revealed that plasma HDL is decreased in SSc-PF and IPF. Moreover, total serum HDL particles have been negatively correlated with mortality or the necessity for lung transplantation in IPF patients [78]. In contrast to HDL, LDL levels are elevated in PF. We have clarified the role of LDL in PF by acting on apoptosis in endothelial and AT2 cells, and activation of fibroblasts. Chylomicrons (CMs) and other lipoproteins have not been studied in PF, neither the changes nor the roles. The uptake and utilization of these lipoproteins are involved in multiple receptors and enzymes; thereby, the whole metabolic processes in fibrotic lungs are demanded. Considering that lipoproteins are primarily produced by extrapulmonary tissues, such as the liver and the intestine, the contribution of these organs to PF, and the regulatory network between lung and extrapulmonary tissues are needed to investigate further.

4. Strengths and Limitations

The reconstructed lipid metabolite profiles in ECs, AT2 cells, 54 macrophages, and fibroblasts contribute to collagen deposition and the lung architecture remodeling observed in PF through various mechanisms. Single-cell RNA-sequencing reanalysis helps to further clarify cell type-specific metabolism changes in PF. Moreover,

uncovering the function of the metabolite-metabolic gene axis in specific cell types is useful for explaining the dysfunction of these cells and, eventually fibrosis. We suggest that abnormal lipid metabolism may be a strong risk factor for PF. When individuals are exposed to any external or internal stimulus, these lipid metabolism disorders may exacerbate the process of PF.

Single-cell metabolomics can more accurately reflect the level of single-cell metabolism in PF than transcriptomics or proteomics. However, at present, only single-cell transcriptomics is being studied in PF research, and there is no single-cell proteome or single-cell metabolome. In addition, many metabolic gene changes at the cellular transcriptional level align well with the metabolomic results of PF tissues or blood, suggesting that the single-cell transcriptome is powerful in revealing metabolism. In many other areas of disease research, single-cell transcription is also used to summarise single-cell metabolism [122-124]. In conclusion, single-cell transcription can reflect, at least in part, the level of single-cell metabolism. Changes in lipid metabolism in other cell types also need to be investigated, in particular, bronchioalveolar stem cells (BASCs) located at the junction of bronchioalveolar [125]. Depending on the location of the injury, BASCs can differentiate unidirectionally into airway epithelial cells or alveolar epithelial cells, playing a role in lung regeneration and the management of PF [126, 127]. Moreover, lipid metabolism has an impact on stem cell function through the induction of fatty acid oxidation [128, 129]. Therefore,

understanding the regulatory network of metabolic programs in BASCs is necessary to clinically restore tissue homeostasis post-injury by manipulating the regenerative machinery.

5. Conclusion

This review summarizes the various lipid species' metabolite changes and reanalyzes their corresponding lipid metabolomic genes at the single-cell level found in PF. It sheds light on the pathogenesis of PF from the perspective of abnormal lipid metabolism and identifies potential targets. Consistently, recent articles have also demonstrated that the important role of metabolism in pulmonary fibrosis from the genomic data, pathogenesis data and reports of pulmonary fibrosis[130-138]. Abnormal lipid metabolism is strongly associated with FVC%, DLCO%, disease severity, progression and survival in PF [16, 34, 56, 139]. It seems that PF can also be viewed as a disease due to abnormal lipid metabolism induced by risk exposures. In patient care, it is imperative to assess both undernutrition and overnutrition in PF patients [140]. The wide spectrum of disrupted lipid metabolism in PF necessitates a precise approach that considers interventions specific to cell types, disease stages, and nutrient cycling. Collectively, these results highlight the clinical relevance of metabolic expression and provide an informative metric for the care of patients.

6. Datasets reanalysis and Statistical analysis

Public scRNA-seq datasets reanalyzed in this study can be found on GEO using accession numbers GSE135893 and GSE136831. According to the cell numbers, data in endothelial and AT2 cells were derived from dataset GSE135893, and data in fibroblasts and macrophages were derived from GSE136831. For normally distributed parameters, the independent sample t-test was employed in cases of homogenous variance; if not, the non-parametric Mann-Whitney test was utilized. A P value of less than 0.05 was considered statistically significant. Except for the DEGs related to lipid metabolism, other DEGs were listed in **Supplemental Table 1**.

7. Conflict of interest

None.

8. Data availability statement

Data available upon request.

9. Author contributions

XS, JC and JW designed the study concept and prepared the main manuscript text; LH and JD were in charge of revising the manuscript; YC, MS and FG prepared figures and tables. WW, DW, CS, XX, NS and XC sumarrized the published literature. YY and CL analysed and interpreted the data. All authors reviewed the manuscript.

10. Acknowledgement and Funding declaration

This work was supported by National Natural Science Foundation of China (82270070, 82030003, 81903203); National Key Research and Development Program of China (2023YFC2507100); the CAMS Innovation Fund for Medical Sciences (2019-I2M-5–066), the Shanghai Municipal Science and Technology Major Project (2023SHZDZX02); Science and Technology Innovation Plan-Basic Research Projects, 20JC1417300, and the 111 Project (B13016).

Reference：

1. Jee AS, Sahhar J, Youssef P, Bleasel J, Adelstein S, Nguyen M, Corte TJ: **Review: Serum biomarkers in idiopathic pulmonary fibrosis and systemic sclerosis associated interstitial lung disease - frontiers and horizons.** *Pharmacol Ther* 2019, **202:**40-52.
2. Hutchinson J, Fogarty A, Hubbard R, McKeever T: **Global incidence and mortality of idiopathic pulmonary fibrosis: a systematic review.** *Eur Respir J* 2015, **46:**795-806.
3. Khanna D, Tashkin DP, Denton CP, Renzoni EA, Desai SR, Varga J: **Etiology, Risk Factors, and Biomarkers in Systemic Sclerosis with Interstitial Lung Disease.** *Am J Respir Crit Care Med* 2020, **201:**650-660.
4. Liu X, Jiang Q, Wu P, Han L, Zhou P: **Global incidence, prevalence and disease burden of silicosis: 30 years' overview and forecasted trends.** *BMC Public Health* 2023, **23:**1366.
5. Alysandratos KD, Russo SJ, Petcherski A, Taddeo EP, Acin-Perez R, Villacorta-Martin C, Jean JC, Mulugeta S, Rodriguez LR, Blum BC, et al: **Patient-specific iPSCs carrying an SFTPC mutation reveal the intrinsic alveolar epithelial dysfunction at the inception of interstitial lung disease.** *Cell Rep* 2021, **36:**109636.
6. Stancil IT, Michalski JE, Davis-Hall D, Chu HW, Park JA, Magin CM, Yang IV, Smith BJ, Dobrinskikh E, Schwartz DA: **Pulmonary fibrosis distal airway epithelia are dynamically and structurally dysfunctional.** *Nat Commun* 2021, **12:**4566.
7. Wang L, Chen R, Li G, Wang Z, Liu J, Liang Y, Liu JP: **FBW7 Mediates Senescence and Pulmonary Fibrosis through Telomere Uncapping.** *Cell Metab* 2020, **32:**860-877 e869.
8. Katzen J, Beers MF: **Contributions of alveolar epithelial cell quality control to pulmonary fibrosis.** *J Clin Invest* 2020, **130:**5088-5099.
9. Cao Z, Lis R, Ginsberg M, Chavez D, Shido K, Rabbany SY, Fong GH, Sakmar TP, Rafii S, Ding BS: **Targeting of the pulmonary capillary vascular niche promotes lung alveolar repair and ameliorates fibrosis.** *Nat Med* 2016, **22:**154-162.
10. Scruggs AM, Grabauskas G, Huang SK: **The Role of KCNMB1 and BK Channels in Myofibroblast Differentiation and Pulmonary Fibrosis.** *Am J Respir Cell Mol Biol* 2020, **62:**191-203.
11. Moss BJ, Ryter SW, Rosas IO: **Pathogenic Mechanisms Underlying Idiopathic Pulmonary Fibrosis.** *Annual Review of Pathology-Mechanisms of Disease* 2022, **17:**515-546.
12. Dempsey TM, Payne S, Sangaralingham L, Yao X, Shah ND, Limper AH: **Adoption of the Antifibrotic Medications Pirfenidone and Nintedanib for Patients with Idiopathic Pulmonary Fibrosis.** *Ann Am Thorac Soc* 2021, **18:**1121-1128.
13. Shi X, Chen Y, Liu Q, Mei X, Liu J, Tang Y, Luo R, Sun D, Ma Y, Wu W, et al: **LDLR dysfunction induces LDL accumulation and promotes pulmonary fibrosis.** *Clin Transl Med* 2022, **12:**e711.
14. Milara J, Navarro R, Juan G, Peiro T, Serrano A, Ramon M, Morcillo E, Cortijo J: **Sphingosine-1-phosphate is increased in patients with idiopathic pulmonary fibrosis and mediates epithelial to mesenchymal transition.** *Thorax* 2012, **67:**147-156.
15. Huang LS, Sudhadevi T, Fu P, Punathil-Kannan PK, Ebenezer DL, Ramchandran R, Putherickal V,

Cheresh P, Zhou G, Ha AW, et al: **Sphingosine Kinase 1/S1P Signaling Contributes to Pulmonary Fibrosis by Activating Hippo/YAP Pathway and Mitochondrial Reactive Oxygen Species in Lung Fibroblasts.** *Int J Mol Sci* 2020, **21**.

16. Huang LS, Berdyshev EV, Tran JT, Xie L, Chen J, Ebenezer DL, Mathew B, Gorshkova I, Zhang W, Reddy SP, et al: **Sphingosine-1-phosphate lyase is an endogenous suppressor of pulmonary fibrosis: role of S1P signalling and autophagy.** *Thorax* 2015, **70:**1138-1148.
17. Zhao J, Okamoto Y, Asano Y, Ishimaru K, Aki S, Yoshioka K, Takuwa N, Wada T, Inagaki Y, Takahashi C, et al: **Sphingosine-1-phosphate receptor-2 facilitates pulmonary fibrosis through potentiating IL-13 pathway in macrophages.** *PLoS One* 2018, **13:**e0197604.
18. Summer R, Mora AL: **Lipid Metabolism: A New Player in the Conundrum of Lung Fibrosis.** *Am J Respir Cell Mol Biol* 2019, **61:**669-670.
19. Wang H, Zhou S, Liu Y, Yu Y, Xu S, Peng L, Ni C: **Exploration study on serum metabolic profiles of Chinese male patients with artificial stone silicosis, silicosis, and coal worker's pneumoconiosis.** *Toxicol Lett* 2022, **356:**132-142.
20. Dalleywater W, Powell HA, Hubbard RB, Navaratnam V: **Risk factors for cardiovascular disease in people with idiopathic pulmonary fibrosis: a population-based study.** *Chest* 2015, **147:**150-156.
21. Guo X, Sunil C, Qian G: **Obesity and the Development of Lung Fibrosis.** *Front Pharmacol* 2021, **12:**812166.
22. King TE, Jr., Pardo A, Selman M: **Idiopathic pulmonary fibrosis.** *Lancet* 2011, **378:**1949-1961.
23. Chung KP, Hsu CL, Fan LC, Huang Z, Bhatia D, Chen YJ, Hisata S, Cho SJ, Nakahira K, Imamura M, et al: **Mitofusins regulate lipid metabolism to mediate the development of lung fibrosis.** *Nat Commun* 2019, **10:**3390.
24. Martin M, Zhang J, Miao Y, He M, Kang J, Huang HY, Chou CH, Huang TS, Hong HC, Su SH, et al: **Role of endothelial cells in pulmonary fibrosis via SREBP2 activation.** *JCI Insight* 2021, **6**.
25. Mould KJ, Moore CM, McManus SA, McCubbrey AL, McClendon JD, Griesmer CL, Henson PM, Janssen WJ: **Airspace Macrophages and Monocytes Exist in Transcriptionally Distinct Subsets in Healthy Adults.** *Am J Respir Crit Care Med* 2021, **203:**946-956.
26. Colin S, Chinetti-Gbaguidi G, Staels B: **Macrophage phenotypes in atherosclerosis.** *Immunol Rev* 2014, **262:**153-166.
27. Cheng P, Li S, Chen H: **Macrophages in Lung Injury, Repair, and Fibrosis.** *Cells* 2021, **10**.
28. Aran D, Looney AP, Liu L, Wu E, Fong V, Hsu A, Chak S, Naikawadi RP, Wolters PJ, Abate AR, et al: **Reference-based analysis of lung single-cell sequencing reveals a transitional profibrotic macrophage.** *Nat Immunol* 2019, **20:**163-172.
29. Valenzi E, Bulik M, Tabib T, Morse C, Sembrat J, Trejo Bittar H, Rojas M, Lafyatis R: **Single-cell analysis reveals fibroblast heterogeneity and myofibroblasts in systemic sclerosis-associated interstitial lung disease.** *Ann Rheum Dis* 2019, **78:**1379-1387.
30. Reyfman PA, Walter JM, Joshi N, Anekalla KR, McQuattie-Pimentel AC, Chiu S, Fernandez R, Akbarpour M, Chen CI, Ren Z, et al: **Single-Cell Transcriptomic Analysis of Human Lung Provides Insights into the Pathobiology of Pulmonary Fibrosis.** *Am J Respir Crit Care Med*

2019, **199:**1517-1536.
31. Habermann AC, Gutierrez AJ, Bui LT, Yahn SL, Winters NI, Calvi CL, Peter L, Chung MI, Taylor CJ, Jetter C, et al: **Single-cell RNA sequencing reveals profibrotic roles of distinct epithelial and mesenchymal lineages in pulmonary fibrosis.** *Sci Adv* 2020, **6:**eaba1972.
32. Adams TS, Schupp JC, Poli S, Ayaub EA, Neumark N, Ahangari F, Chu SG, Raby BA, DeIuliis G, Januszyk M, et al: **Single-cell RNA-seq reveals ectopic and aberrant lung-resident cell populations in idiopathic pulmonary fibrosis.** *Sci Adv* 2020, **6:**eaba1983.
33. Larson-Casey JL, Deshane JS, Ryan AJ, Thannickal VJ, Carter AB: **Macrophage Akt1 Kinase-Mediated Mitophagy Modulates Apoptosis Resistance and Pulmonary Fibrosis.** *Immunity* 2016, **44:**582-596.
34. Tlatelpa-Romero B, Cazares-Ordonez V, Oyarzabal LF, Vazquez-de-Lara LG: **The Role of Pulmonary Surfactant Phospholipids in Fibrotic Lung Diseases.** *Int J Mol Sci* 2022, **24**.
35. Yan F, Wen Z, Wang R, Luo W, Du Y, Wang W, Chen X: **Identification of the lipid biomarkers from plasma in idiopathic pulmonary fibrosis by Lipidomics.** *BMC Pulm Med* 2017, **17:**174.
36. Carter CL, Jones JW, Farese AM, MacVittie TJ, Kane MA: **Lipidomic dysregulation within the lung parenchyma following whole-thorax lung irradiation: Markers of injury, inflammation and fibrosis detected by MALDI-MSI.** *Sci Rep* 2017, **7:**10343.
37. Nambiar S, Clynick B, How BS, King A, Walters EH, Goh NS, Corte TJ, Trengove R, Tan D, Moodley Y: **There is detectable variation in the lipidomic profile between stable and progressive patients with idiopathic pulmonary fibrosis (IPF).** *Respir Res* 2021, **22:**105.
38. Vazquez-de-Lara LG, Tlatelpa-Romero B, Romero Y, Fernandez-Tamayo N, Vazquez-de-Lara F, J MJ-J, Garcia-Carrasco M, de-la-Rosa Paredes R, Cisneros-Lira JG, Mendoza-Milla C, et al: **Phosphatidylethanolamine Induces an Antifibrotic Phenotype in Normal Human Lung Fibroblasts and Ameliorates Bleomycin-Induced Lung Fibrosis in Mice.** *Int J Mol Sci* 2018, **19**.
39. Preuss S, Scheiermann J, Stadelmann S, Omam FD, Winoto-Morbach S, Lex D, von Bismarck P, Adam-Klages S, Knerlich-Lukoschus F, Wesch D, et al: **18:1/18:1-Dioleoyl-phosphatidylglycerol prevents alveolar epithelial apoptosis and profibrotic stimulus in a neonatal piglet model of acute respiratory distress syndrome.** *Pulm Pharmacol Ther* 2014, **28:**25-34.
40. Shea BS, Tager AM: **Role of the lysophospholipid mediators lysophosphatidic acid and sphingosine 1-phosphate in lung fibrosis.** *Proc Am Thorac Soc* 2012, **9:**102-110.
41. Romero F, Shah D, Duong M, Penn RB, Fessler MB, Madenspacher J, Stafstrom W, Kavuru M, Lu B, Kallen CB, et al: **A pneumocyte-macrophage paracrine lipid axis drives the lung toward fibrosis.** *Am J Respir Cell Mol Biol* 2015, **53:**74-86.
42. Gong Y, Wang N, Liu N, Dong H: **Lipid Peroxidation and GPX4 Inhibition Are Common Causes for Myofibroblast Differentiation and Ferroptosis.** *DNA Cell Biol* 2019, **38:**725-733.
43. Tao N, Li K, Liu J: **Molecular Mechanisms of Ferroptosis and Its Role in Pulmonary Disease.** *Oxid Med Cell Longev* 2020, **2020:**9547127.
44. Xu W, Deng H, Hu S, Zhang Y, Zheng L, Liu M, Chen Y, Wei J, Yang H, Lv X: **Role of Ferroptosis in Lung Diseases.** *J Inflamm Res* 2021, **14:**2079-2090.

45. Chu SG, Villalba JA, Liang X, Xiong K, Tsoyi K, Ith B, Ayaub EA, Tatituri RV, Byers DE, Hsu FF, et al: **Palmitic Acid-Rich High-Fat Diet Exacerbates Experimental Pulmonary Fibrosis by Modulating Endoplasmic Reticulum Stress.** *Am J Respir Cell Mol Biol* 2019, **61:**737-746.

46. Wang X, Chen Y, Lv L, Chen J: **Silencing CD36 gene expression results in the inhibition of latent-TGF-beta1 activation and suppression of silica-induced lung fibrosis in the rat.** *Respir Res* 2009, **10:**36.

47. McGowan SE, Torday JS: **The pulmonary lipofibroblast (lipid interstitial cell) and its contributions to alveolar development.** *Annu Rev Physiol* 1997, **59:**43-62.

48. Chao CM, El Agha E, Tiozzo C, Minoo P, Bellusci S: **A breath of fresh air on the mesenchyme: impact of impaired mesenchymal development on the pathogenesis of bronchopulmonary dysplasia.** *Front Med (Lausanne)* 2015, **2:**27.

49. Torday JS, Rehan VK: **Stretch-stimulated surfactant synthesis is coordinated by the paracrine actions of PTHrP and leptin.** *Am J Physiol Lung Cell Mol Physiol* 2002, **283:**L130-135.

50. Kumar A, Sundaram K, Mu J, Dryden GW, Sriwastva MK, Lei C, Zhang L, Qiu X, Xu F, Yan J, et al: **High-fat diet-induced upregulation of exosomal phosphatidylcholine contributes to insulin resistance.** *Nat Commun* 2021, **12:**213.

51. Teichgraber V, Ulrich M, Endlich N, Riethmuller J, Wilker B, De Oliveira-Munding CC, van Heeckeren AM, Barr ML, von Kurthy G, Schmid KW, et al: **Ceramide accumulation mediates inflammation, cell death and infection susceptibility in cystic fibrosis.** *Nat Med* 2008, **14:**382-391.

52. Hoefer J, Azam MA, Kroetsch JT, Leong-Poi H, Momen MA, Voigtlaender-Bolz J, Scherer EQ, Meissner A, Bolz SS, Husain M: **Sphingosine-1-phosphate-dependent activation of p38 MAPK maintains elevated peripheral resistance in heart failure through increased myogenic vasoconstriction.** *Circ Res* 2010, **107:**923-933.

53. Bienias K, Fiedorowicz A, Sadowska A, Prokopiuk S, Car H: **Regulation of sphingomyelin metabolism.** *Pharmacol Rep* 2016, **68:**570-581.

54. Tibboel J, Reiss I, de Jongste JC, Post M: **Sphingolipids in lung growth and repair.** *Chest* 2014, **145:**120-128.

55. Zhao YD, Yin L, Archer S, Lu C, Zhao G, Yao Y, Wu L, Hsin M, Waddell TK, Keshavjee S, et al: **Metabolic heterogeneity of idiopathic pulmonary fibrosis: a metabolomic study.** *BMJ Open Respir Res* 2017, **4:**e000183.

56. Huang LS, Berdyshev E, Mathew B, Fu P, Gorshkova IA, He D, Ma W, Noth I, Ma SF, Pendyala S, et al: **Targeting sphingosine kinase 1 attenuates bleomycin-induced pulmonary fibrosis.** *FASEB J* 2013, **27:**1749-1760.

57. Park SJ, Im DS: **Deficiency of Sphingosine-1-Phosphate Receptor 2 (S1P2) Attenuates Bleomycin-Induced Pulmonary Fibrosis.** *Biomol Ther (Seoul)* 2019, **27:**318-326.

58. Takuwa Y, Ikeda H, Okamoto Y, Takuwa N, Yoshioka K: **Sphingosine-1-phosphate as a mediator involved in development of fibrotic diseases.** *Biochim Biophys Acta* 2013, **1831:**185-192.

59. Knipe RS, Spinney JJ, Abe EA, Probst CK, Franklin A, Logue A, Giacona F, Drummond M,

Griffith J, Brazee PL, et al: **Endothelial-Specific Loss of Sphingosine-1-Phosphate Receptor 1 Increases Vascular Permeability and Exacerbates Bleomycin-induced Pulmonary Fibrosis.** *Am J Respir Cell Mol Biol* 2022, **66:**38-52.

60. Kimura T, Ishii Y, Morishima Y, Shibuya A, Shibuya K, Taniguchi M, Mochizuki M, Hegab AE, Sakamoto T, Nomura A, Sekizawa K: **Treatment with alpha-galactosylceramide attenuates the development of bleomycin-induced pulmonary fibrosis.** *J Immunol* 2004, **172:**5782-5789.
61. Fireman E, Spitzer S, Grief J, Kivity S, Topilsky M: **Cholesterol crystals in BAL fluid from patients with idiopathic pulmonary fibrosis.** *Respir Med* 1996, **90:**361-363.
62. Ichikawa T, Sugiura H, Koarai A, Kikuchi T, Hiramatsu M, Kawabata H, Akamatsu K, Hirano T, Nakanishi M, Matsunaga K, et al: **25-hydroxycholesterol promotes fibroblast-mediated tissue remodeling through NF-kappaB dependent pathway.** *Exp Cell Res* 2013, **319:**1176-1186.
63. Luo J, Yang H, Song BL: **Mechanisms and regulation of cholesterol homeostasis.** *Nat Rev Mol Cell Biol* 2020, **21:**225-245.
64. Shichino S, Ueha S, Hashimoto S, Otsuji M, Abe J, Tsukui T, Deshimaru S, Nakajima T, Kosugi-Kanaya M, Shand FH, et al: **Transcriptome network analysis identifies protective role of the LXR/SREBP-1c axis in murine pulmonary fibrosis.** *JCI Insight* 2019, **4**.
65. Romero F, Hong X, Shah D, Kallen CB, Rosas I, Guo Z, Schriner D, Barta J, Shaghaghi H, Hoek JB, et al: **Lipid Synthesis Is Required to Resolve Endoplasmic Reticulum Stress and Limit Fibrotic Responses in the Lung.** *Am J Respir Cell Mol Biol* 2018, **59:**225-236.
66. Fernandez-Suarez ME, Daimiel L, Villa-Turegano G, Pavon MV, Busto R, Escola-Gil JC, Platt FM, Lasuncion MA, Martinez-Botas J, Gomez-Coronado D: **Selective estrogen receptor modulators (SERMs) affect cholesterol homeostasis through the master regulators SREBP and LXR.** *Biomed Pharmacother* 2021, **141:**111871.
67. Gowdy KM, Fessler MB: **Emerging roles for cholesterol and lipoproteins in lung disease.** *Pulm Pharmacol Ther* 2013, **26:**430-437.
68. Yao X, Gordon EM, Figueroa DM, Barochia AV, Levine SJ: **Emerging Roles of Apolipoprotein E and Apolipoprotein A-I in the Pathogenesis and Treatment of Lung Disease.** *Am J Respir Cell Mol Biol* 2016, **55:**159-169.
69. Goldstein JL, Brown MS, Anderson RG, Russell DW, Schneider WJ: **Receptor-mediated endocytosis: concepts emerging from the LDL receptor system.** *Annu Rev Cell Biol* 1985, **1:**1-39.
70. Voyno-Yasenetskaya TA, Dobbs LG, Erickson SK, Hamilton RL: **Low density lipoprotein- and high density lipoprotein-mediated signal transduction and exocytosis in alveolar type II cells.** *Proc Natl Acad Sci U S A* 1993, **90:**4256-4260.
71. Massaro D, Massaro GD: **Developmental alveologenesis: new roles for ApoE and LDL receptor.** *Pediatr Res* 2011, **70:**458-461.
72. Aihara K, Handa T, Nagai S, Tanizawa K, Ikezoe K, Watanabe K, Chihara Y, Harada Y, Yoshimura C, Oga T, et al: **Impaired endothelium-dependent vasodilator response in patients with pulmonary fibrosis.** *Respir Med* 2013, **107:**269-275.

73. Kotlyarov S: **Participation of ABCA1 Transporter in Pathogenesis of Chronic Obstructive Pulmonary Disease.** *Int J Mol Sci* 2021, **22**.

74. Brunham LR, Kruit JK, Iqbal J, Fievet C, Timmins JM, Pape TD, Coburn BA, Bissada N, Staels B, Groen AK, et al: **Intestinal ABCA1 directly contributes to HDL biogenesis in vivo.** *J Clin Invest* 2006, **116:**1052-1062.

75. Barter PJ, Brewer HB, Jr., Chapman MJ, Hennekens CH, Rader DJ, Tall AR: **Cholesteryl ester transfer protein: a novel target for raising HDL and inhibiting atherosclerosis.** *Arterioscler Thromb Vasc Biol* 2003, **23:**160-167.

76. Chang TY, Chang CC, Ohgami N, Yamauchi Y: **Cholesterol sensing, trafficking, and esterification.** *Annu Rev Cell Dev Biol* 2006, **22:**129-157.

77. Venosa A, Smith LC, Murray A, Banota T, Gow AJ, Laskin JD, Laskin DL: **Regulation of Macrophage Foam Cell Formation During Nitrogen Mustard (NM)-Induced Pulmonary Fibrosis by Lung Lipids.** *Toxicol Sci* 2019, **172:**344-358.

78. Barochia AV, Kaler M, Weir N, Gordon EM, Figueroa DM, Yao X, Lemma WoldeHanna M, Sampson M, Remaley AT, Grant G, et al: **Serum levels of small HDL particles are negatively correlated with death or lung transplantation in an observational study of idiopathic pulmonary fibrosis.** *Eur Respir J* 2021, **58**.

79. Nissen SE, Tuzcu EM, Brewer HB, Sipahi I, Nicholls SJ, Ganz P, Schoenhagen P, Waters DD, Pepine CJ, Crowe TD, et al: **Effect of ACAT inhibition on the progression of coronary atherosclerosis.** *N Engl J Med* 2006, **354:**1253-1263.

80. Huang B, Song BL, Xu C: **Cholesterol metabolism in cancer: mechanisms and therapeutic opportunities.** *Nat Metab* 2020, **2:**132-141.

81. Montesinos J, Pera M, Larrea D, Guardia-Laguarta C, Agrawal RR, Velasco KR, Yun TD, Stavrovskaya IG, Xu Y, Koo SY, et al: **The Alzheimer's disease-associated C99 fragment of APP regulates cellular cholesterol trafficking.** *EMBO J* 2020, **39:**e103791.

82. Comeglio P, Morelli A, Adorini L, Maggi M, Vignozzi L: **Beneficial effects of bile acid receptor agonists in pulmonary disease models.** *Expert Opin Investig Drugs* 2017, **26:**1215-1228.

83. Sun L, Cai J, Gonzalez FJ: **The role of farnesoid X receptor in metabolic diseases, and gastrointestinal and liver cancer.** *Nat Rev Gastroenterol Hepatol* 2021, **18:**335-347.

84. Woods DF, Flynn S, Caparros-Martin JA, Stick SM, Reen FJ, O'Gara F: **Systems Biology and Bile Acid Signalling in Microbiome-Host Interactions in the Cystic Fibrosis Lung.** *Antibiotics (Basel)* 2021, **10**.

85. Manni ML, Heinrich VA, Buchan GJ, O'Brien JP, Uvalle C, Cechova V, Koudelka A, Ukani D, Rawas-Qalaji M, Oury TD, et al: **Nitroalkene fatty acids modulate bile acid metabolism and lung function in obese asthma.** *Sci Rep* 2021, **11:**17788.

86. Zhao YD, Yun HZH, Peng J, Yin L, Chu L, Wu L, Michalek R, Liu M, Keshavjee S, Waddell T, et al: **De novo synthesize of bile acids in pulmonary arterial hypertension lung.** *Metabolomics* 2014, **10:**1169-1175.

87. Chen B, Cai HR, Xue S, You WJ, Liu B, Jiang HD: **Bile acids induce activation of alveolar epithelial cells and lung fibroblasts through farnesoid X receptor-dependent and**

independent pathways. *Respirology* 2016, **21:**1075-1080.
88. Chen B, You WJ, Liu XQ, Xue S, Qin H, Jiang HD: **Chronic microaspiration of bile acids induces lung fibrosis through multiple mechanisms in rats.** *Clin Sci (Lond)* 2017, **131:**951-963.
89. Zhou J, Huang N, Guo Y, Cui S, Ge C, He Q, Pan X, Wang G, Wang H, Hao H: **Combined obeticholic acid and apoptosis inhibitor treatment alleviates liver fibrosis.** *Acta Pharm Sin B* 2019, **9:**526-536.
90. Gadaleta RM, van Erpecum KJ, Oldenburg B, Willemsen EC, Renooij W, Murzilli S, Klomp LW, Siersema PD, Schipper ME, Danese S, et al: **Farnesoid X receptor activation inhibits inflammation and preserves the intestinal barrier in inflammatory bowel disease.** *Gut* 2011, **60:**463-472.
91. Vignozzi L, Morelli A, Cellai I, Filippi S, Comeglio P, Sarchielli E, Maneschi E, Vannelli GB, Adorini L, Maggi M: **Cardiopulmonary protective effects of the selective FXR agonist obeticholic acid in the rat model of monocrotaline-induced pulmonary hypertension.** *J Steroid Biochem Mol Biol* 2017, **165:**277-292.
92. Comeglio P, Filippi S, Sarchielli E, Morelli A, Cellai I, Corcetto F, Corno C, Maneschi E, Pini A, Adorini L, et al: **Anti-fibrotic effects of chronic treatment with the selective FXR agonist obeticholic acid in the bleomycin-induced rat model of pulmonary fibrosis.** *J Steroid Biochem Mol Biol* 2017, **168:**26-37.
93. Raghu G, Selman M: **Nintedanib and pirfenidone. New antifibrotic treatments indicated for idiopathic pulmonary fibrosis offer hopes and raises questions.** *Am J Respir Crit Care Med* 2015, **191:**252-254.
94. Morani A, Barros RP, Imamov O, Hultenby K, Arner A, Warner M, Gustafsson JA: **Lung dysfunction causes systemic hypoxia in estrogen receptor beta knockout (ERbeta-/-) mice.** *Proc Natl Acad Sci U S A* 2006, **103:**7165-7169.
95. Elliot S, Periera-Simon S, Xia X, Catanuto P, Rubio G, Shahzeidi S, El Salem F, Shapiro J, Briegel K, Korach KS, Glassberg MK: **MicroRNA let-7 Downregulates Ligand-Independent Estrogen Receptor-mediated Male-Predominant Pulmonary Fibrosis.** *Am J Respir Crit Care Med* 2019, **200:**1246-1257.
96. Mehrad M, Trejo Bittar HE, Yousem SA: **Sex steroid receptor expression in idiopathic pulmonary fibrosis.** *Hum Pathol* 2017, **66:**200-205.
97. Mendoza-Milla C, Valero Jimenez A, Rangel C, Lozano A, Morales V, Becerril C, Chavira R, Ruiz V, Barrera L, Montano M, et al: **Dehydroepiandrosterone has strong antifibrotic effects and is decreased in idiopathic pulmonary fibrosis.** *Eur Respir J* 2013, **42:**1309-1321.
98. Voltz JW, Card JW, Carey MA, Degraff LM, Ferguson CD, Flake GP, Bonner JC, Korach KS, Zeldin DC: **Male sex hormones exacerbate lung function impairment after bleomycin-induced pulmonary fibrosis.** *Am J Respir Cell Mol Biol* 2008, **39:**45-52.
99. Fang C, Huang H, Zhang Q, Wang N, Jing X, Guo J, Ferianc M, Xu Z: **Relation between sex hormones and leucocyte telomere length in men with idiopathic pulmonary fibrosis.** *Respirology* 2020, **25:**1265-1273.
100. Li SR, Tan ZX, Chen YH, Hu B, Zhang C, Wang H, Zhao H, Xu DX: **Vitamin D deficiency**

exacerbates bleomycin-induced pulmonary fibrosis partially through aggravating TGF-beta/Smad2/3-mediated epithelial-mesenchymal transition. *Respir Res* 2019, **20:**266.

101. Zhu W, Ding Q, Wang L, Xu G, Diao Y, Qu S, Chen S, Shi Y: **Vitamin D3 alleviates pulmonary fibrosis by regulating the MAPK pathway via targeting PSAT1 expression in vivo and in vitro.** *Int Immunopharmacol* 2021, **101:**108212.
102. Tzilas V, Bouros E, Barbayianni I, Karampitsakos T, Kourtidou S, Ntassiou M, Ninou I, Aidinis V, Bouros D, Tzouvelekis A: **Vitamin D prevents experimental lung fibrosis and predicts survival in patients with idiopathic pulmonary fibrosis.** *Pulm Pharmacol Ther* 2019, **55:**17-24.
103. Naccache JM, Jouneau S, Didier M, Borie R, Cachanado M, Bourdin A, Reynaud-Gaubert M, Bonniaud P, Israel-Biet D, Prevot G, et al: **Cyclophosphamide added to glucocorticoids in acute exacerbation of idiopathic pulmonary fibrosis (EXAFIP): a randomised, double-blind, placebo-controlled, phase 3 trial.** *Lancet Respir Med* 2022, **10:**26-34.
104. Zhang HQ, Yau YF, Wong MS, Man OY, He YY, Chan N, Li M: **Chinese medicine formula DSQRL versus glucocorticoids for the treatment of experimental pulmonary fibrosis.** *J Ethnopharmacol* 2008, **116:**318-324.
105. Wen FQ, Kohyama T, Skold CM, Zhu YK, Liu X, Romberger DJ, Stoner J, Rennard SI: **Glucocorticoids modulate TGF-beta production.** *Inflammation* 2002, **26:**279-290.
106. Ozaki T, Nakayama T, Ishimi H, Kawano T, Yasuoka S, Tsubura E: **Glucocorticoid receptors in bronchoalveolar cells from patients with idiopathic pulmonary fibrosis.** *Am Rev Respir Dis* 1982, **126:**968-971.
107. Pujols L, Xaubet A, Ramirez J, Mullol J, Roca-Ferrer J, Torrego A, Cidlowski JA, Picado C: **Expression of glucocorticoid receptors alpha and beta in steroid sensitive and steroid insensitive interstitial lung diseases.** *Thorax* 2004, **59:**687-693.
108. Barrera-Chimal J, Jaisser F: **MR (Mineralocorticoid Receptor) in Endothelial Cells: A Major Contributor in Pulmonary Arterial Hypertension Remodeling.** *Hypertension* 2021, **78:**466-468.
109. Preston IR, Sagliani KD, Warburton RR, Hill NS, Fanburg BL, Jaffe IZ: **Mineralocorticoid receptor antagonism attenuates experimental pulmonary hypertension.** *Am J Physiol Lung Cell Mol Physiol* 2013, **304:**L678-688.
110. Kotfis K, Karolak I, Lechowicz K, Zegan-Baranska M, Pikulska A, Niedzwiedzka-Rystwej P, Kawa M, Sienko J, Szylinska A, Wisniewska M: **Mineralocorticoid Receptor Antagonist (Potassium Canrenoate) Does Not Influence Outcome in the Treatment of COVID-19-Associated Pneumonia and Fibrosis-A Randomized Placebo Controlled Clinical Trial.** *Pharmaceuticals (Basel)* 2022, **15**.
111. Giacomelli C, Piccarducci R, Marchetti L, Romei C, Martini C: **Pulmonary fibrosis from molecular mechanisms to therapeutic interventions: lessons from post-COVID-19 patients.** *Biochem Pharmacol* 2021, **193:**114812.
112. El Agha E, Moiseenko A, Kheirollahi V, De Langhe S, Crnkovic S, Kwapiszewska G, Szibor M, Kosanovic D, Schwind F, Schermuly RT, et al: **Two-Way Conversion between Lipogenic and Myogenic Fibroblastic Phenotypes Marks the Progression and Resolution of Lung Fibrosis.**

Cell Stem Cell 2017, **20:**261-273 e263.

113. Onal G, Kutlu O, Gozuacik D, Dokmeci Emre S: **Lipid Droplets in Health and Disease.** *Lipids Health Dis* 2017, **16:**128.

114. Kheirollahi V, Wasnick RM, Biasin V, Vazquez-Armendariz AI, Chu X, Moiseenko A, Weiss A, Wilhelm J, Zhang JS, Kwapiszewska G, et al: **Metformin induces lipogenic differentiation in myofibroblasts to reverse lung fibrosis.** *Nat Commun* 2019, **10:**2987.

115. Varisco BM, Ambalavanan N, Whitsett JA, Hagood JS: **Thy-1 signals through PPARgamma to promote lipofibroblast differentiation in the developing lung.** *Am J Respir Cell Mol Biol* 2012, **46:**765-772.

116. Geng J, Liu Y, Dai H, Wang C: **Fatty Acid Metabolism and Idiopathic Pulmonary Fibrosis.** *Front Physiol* 2021, **12:**794629.

117. Sunaga H, Matsui H, Ueno M, Maeno T, Iso T, Syamsunarno MR, Anjo S, Matsuzaka T, Shimano H, Yokoyama T, Kurabayashi M: **Deranged fatty acid composition causes pulmonary fibrosis in Elovl6-deficient mice.** *Nat Commun* 2013, **4:**2563.

118. Kim HS, Yoo HJ, Lee KM, Song HE, Kim SJ, Lee JO, Hwang JJ, Song JW: **Stearic acid attenuates profibrotic signalling in idiopathic pulmonary fibrosis.** *Respirology* 2021, **26:**255-263.

119. Guillotin D, Taylor AR, Plate M, Mercer PF, Edwards LM, Haggart R, Miele G, McAnulty RJ, Maher TM, Hynds RE, et al: **Transcriptome analysis of IPF fibroblastic foci identifies key pathways involved in fibrogenesis.** *Thorax* 2021, **76:**73-82.

120. Plate M, Guillotin D, Chambers RC: **The promise of mTOR as a therapeutic target pathway in idiopathic pulmonary fibrosis.** *Eur Respir Rev* 2020, **29**.

121. Paton CM, Ntambi JM: **Biochemical and physiological function of stearoyl-CoA desaturase.** *Am J Physiol Endocrinol Metab* 2009, **297:**E28-37.

122. Wang G, Qiu M, Xing X, Zhou J, Yao H, Li M, Yin R, Hou Y, Li Y, Pan S, et al: **Lung cancer scRNA-seq and lipidomics reveal aberrant lipid metabolism for early-stage diagnosis.** *Sci Transl Med* 2022, **14:**eabk2756.

123. Alghamdi N, Chang W, Dang P, Lu X, Wan C, Gampala S, Huang Z, Wang J, Ma Q, Zang Y, et al: **A graph neural network model to estimate cell-wise metabolic flux using single-cell RNA-seq data.** *Genome Res* 2021, **31:**1867-1884.

124. Huang Y, Mohanty V, Dede M, Tsai K, Daher M, Li L, Rezvani K, Chen K: **Characterizing cancer metabolism from bulk and single-cell RNA-seq data using METAFlux.** *Nat Commun* 2023, **14:**4883.

125. Liu Q, Liu K, Cui G, Huang X, Yao S, Guo W, Qin Z, Li Y, Yang R, Pu W, et al: **Lung regeneration by multipotent stem cells residing at the bronchioalveolar-duct junction.** *Nat Genet* 2019, **51:**728-738.

126. Basil MC, Cardenas-Diaz FL, Kathiriya JJ, Morley MP, Carl J, Brumwell AN, Katzen J, Slovik KJ, Babu A, Zhou S, et al: **Human distal airways contain a multipotent secretory cell that can regenerate alveoli.** *Nature* 2022, **604:**120-126.

127. Ma Q, Ma Y, Dai X, Ren T, Fu Y, Liu W, Han Y, Wu Y, Cheng Y, Zhang T, Zuo W: **Regeneration of functional alveoli by adult human SOX9(+) airway basal cell transplantation.** *Protein Cell*

2018, **9:**267-282.

128. Li X, Wu J, Sun X, Wu Q, Li Y, Li K, Zhang Q, Li Y, Abel ED, Chen H: **Autophagy Reprograms Alveolar Progenitor Cell Metabolism in Response to Lung Injury.** *Stem Cell Reports* 2020, **14:**420-432.

129. Li X, Zhao F, Wang A, Cheng P, Chen H: **Role and mechanisms of autophagy in lung metabolism and repair.** *Cell Mol Life Sci* 2021, **78:**5051-5068.

130. Karampitsakos T, Juan-Guardela BM, Tzouvelekis A, Herazo-Maya JD: **Precision medicine advances in idiopathic pulmonary fibrosis.** *EBioMedicine* 2023, **95:**104766.

131. Ntatsoulis K, Karampitsakos T, Tsitoura E, Stylianaki EA, Matralis AN, Tzouvelekis A, Antoniou K, Aidinis V: **Commonalities Between ARDS, Pulmonary Fibrosis and COVID-19: The Potential of Autotaxin as a Therapeutic Target.** *Front Immunol* 2021, **12:**687397.

132. Tzilas V, Tzouvelekis A, Bouros E, Karampitsakos T, Ntassiou M, Avdoula E, Trachalaki A, Antoniou K, Raghu G, Bouros D: **Clinical experience with antifibrotics in fibrotic hypersensitivity pneumonitis: a 3-year real-life observational study.** *ERJ Open Res* 2020, **6**.

133. Karampitsakos T, Spagnolo P, Mogulkoc N, Wuyts WA, Tomassetti S, Bendstrup E, Molina-Molina M, Manali ED, Unat OS, Bonella F, et al: **Lung cancer in patients with idiopathic pulmonary fibrosis: A retrospective multicentre study in Europe.** *Respirology* 2023, **28:**56-65.

134. Tzouvelekis A, Karampitsakos T, Krompa A, Markozannes E, Bouros D: **False Positive COVID-19 Antibody Test in a Case of Granulomatosis With Polyangiitis.** *Front Med (Lausanne)* 2020, **7:**399.

135. Tzouvelekis A, Karampitsakos T, Kourtidou S, Bouros E, Tzilas V, Katsaras M, Antonou C, Dassiou M, Bouros D: **Impact of Depression on Patients With Idiopathic Pulmonary Fibrosis.** *Front Med (Lausanne)* 2020, **7:**29.

136. Tzouvelekis A, Antoniou K, Kreuter M, Evison M, Blum TG, Poletti V, Grigoriu B, Vancheri C, Spagnolo P, Karampitsakos T, et al: **The DIAMORFOSIS (DIAgnosis and Management Of lung canceR and FibrOSIS) survey: international survey and call for consensus.** *ERJ Open Res* 2021, **7**.

137. Kyriakopoulos C, Gogali A, Exarchos K, Potonos D, Tatsis K, Apollonatou V, Loukides S, Papiris S, Sigala I, Katsaounou P, et al: **Reduction in Hospitalizations for Respiratory Diseases during the First COVID-19 Wave in Greece.** *Respiration* 2021, **100:**588-593.

138. Xylourgidis N, Min K, Ahangari F, Yu G, Herazo-Maya JD, Karampitsakos T, Aidinis V, Binzenhofer L, Bouros D, Bennett AM, et al: **Role of dual-specificity protein phosphatase DUSP10/MKP-5 in pulmonary fibrosis.** *Am J Physiol Lung Cell Mol Physiol* 2019, **317:**L678-L689.

139. Suryadevara V, Ramchandran R, Kamp DW, Natarajan V: **Lipid Mediators Regulate Pulmonary Fibrosis: Potential Mechanisms and Signaling Pathways.** *Int J Mol Sci* 2020, **21**.

140. Faverio P, Bocchino M, Caminati A, Fumagalli A, Gasbarra M, Iovino P, Petruzzi A, Scalfi L, Sebastiani A, Stanziola AA, Sanduzzi A: **Nutrition in Patients with Idiopathic Pulmonary Fibrosis: Critical Issues Analysis and Future Research Directions.** *Nutrients* 2020, **12**.

1

The_novel_molecular_mechanism_of_pulmonary_fibrosis__...

ORIGINALITY REPORT

8%
SIMILARITY INDEX

PRIMARY SOURCES

1	pure.rug.nl<br>Internet	100 words — 1%
2	www.frontiersin.org<br>Internet	51 words — 1%
3	www.mdpi.com<br>Internet	49 words — 1%
4	www.science.gov<br>Internet	27 words — < 1%
5	journals.plos.org<br>Internet	22 words — < 1%
6	"Abstracts of 52nd EASD Annual Meeting", Diabetologia, 2016<br>Crossref	20 words — < 1%
7	Lund, E.D.. "Phospholipid biosynthesis in the oyster protozoan parasite, Perkinsus marinus", Molecular & Biochemical Parasitology, 200205<br>Crossref	20 words — < 1%
8	online.medunigraz.at<br>Internet	17 words — < 1%

9 Benjamin J. Moss, Stefan W. Ryter, Ivan O. Rosas. "Pathogenic Mechanisms Underlying Idiopathic Pulmonary Fibrosis", Annual Review of Pathology: Mechanisms of Disease, 2022 — 16 words — < 1%
Crossref

10 www.ncbi.nlm.nih.gov — 16 words — < 1%
Internet

11 www.scielo.br — 15 words — < 1%
Internet

12 Cortes, Victor A., Dolores Busso, Pablo Mardones, Alberto Maiz, Antonio Arteaga, Flavio Nervi, and Attilio Rigotti. "Advances in the physiological and pathological implications of cholesterol : Cholesterol in health and disease", Biological Reviews, 2013. — 12 words — < 1%
Crossref

13 karger.com — 12 words — < 1%
Internet

14 link.springer.com — 12 words — < 1%
Internet

15 publicatio.bibl.u-szeged.hu — 12 words — < 1%
Internet

16 www.jlr.org — 12 words — < 1%
Internet

17 backend.orbit.dtu.dk — 11 words — < 1%
Internet

18 rcastoragev2.blob.core.windows.net — 11 words — < 1%
Internet

19 www.atsjournals.org
Internet
11 words — < 1%

20 www.biorxiv.org
Internet
11 words — < 1%

21 Surafel Mulugeta, Shin-Ichi Nureki, Michael F. Beers. "Lost after translation: insights from pulmonary surfactant for understanding the role of alveolar epithelial dysfunction and cellular quality control in fibrotic lung disease", American Journal of Physiology-Lung Cellular and Molecular Physiology, 2015
Crossref
10 words — < 1%

22 Xiangguang Shi, Yahui Chen, Qingmei Liu, Xueqian Mei et al. "LDLR dysfunction induces LDL accumulation and promotes pulmonary fibrosis", Clinical and Translational Medicine, 2022
Crossref
10 words — < 1%

23 ruor.uottawa.ca
Internet
10 words — < 1%

24 stemcellres.biomedcentral.com
Internet
10 words — < 1%

25 James D Johnson. "β-cell ABCA1 influences insulin secretion, glucose homeostasis and response to thiazolidinedione treatment", Nature Medicine, 03/2007
Crossref
9 words — < 1%

26 Xiaoping Zhang, Ying Liu, Runxia Shao, Wei Li. "Cdc42-interacting protein 4 silencing relieves pulmonary fibrosis in STZ-induced diabetic mice via the Wnt/GSK-3β/β-catenin pathway", Experimental Cell Research, 2017
Crossref
9 words — < 1%

27 mdpi-res.com
Internet
9 words — < 1%

28 worldwidescience.org
Internet
9 words — < 1%

29 www.esp.org
Internet
9 words — < 1%

30 www.geneontology.org
Internet
9 words — < 1%

31 www.wjgnet.com
Internet
9 words — < 1%

32 "Bile Acids and Their Receptors", Springer Science and Business Media LLC, 2019
Crossref
8 words — < 1%

33 "Idiopathic Pulmonary Fibrosis", Springer Science and Business Media LLC, 2019
Crossref
8 words — < 1%

34 "Sphingolipids in Disease", Springer Science and Business Media LLC, 2013
Crossref
8 words — < 1%

35 Anna Papazoglou, Mengqi Huang, Melissa Bulik, Annika Lafyatis et al. "Epigenetic Regulation of Profibrotic Macrophages in Systemic Sclerosis–Associated Interstitial Lung Disease", Arthritis & Rheumatology, 2022
Crossref
8 words — < 1%

36 Argen Mamazhakypov, Ralph T. Schermuly, Liliana Schaefer, Malgorzata Wygrecka. "Lipids - two sides of the same coin in lung fibrosis", Cellular Signalling, 2019
Crossref
8 words — < 1%

37 Carman, George M., and Gil-Soo Han. "Regulation of Phospholipid Synthesis in the Yeast <i>Saccharomyces cerevisiae</i>", Annual Review of Biochemistry, 2011.
Crossref

8 words — < 1%

38 Ha, Alison Wing-Zi. "Aberrant Autotaxin/Lysophosphatidic Acid Signaling in the Pathogenesis of Bronchopulmonary Dysplasia", University of Illinois at Chicago, 2023
ProQuest

8 words — < 1%

39 Hyun Ju Lee, Youn Jin Lee, Chang Won Choi, Jin-A Lee, Ee-Kyung Kim, Han-Suk Kim, Beyong Il Kim, Jung-Hwan Choi. "Rosiglitazone, a Peroxisome Proliferator-Activated Receptor-γ Agonist, Restores Alveolar and Pulmonary Vascular Development in a Rat Model of Bronchopulmonary Dysplasia", Yonsei Medical Journal, 2014
Crossref

8 words — < 1%

40 Manyu Zhao, Liqun Wang, Mengzhu Wang, Shijie Zhou et al. "Targeting fibrosis: mechanisms and clinical trials", Signal Transduction and Targeted Therapy, 2022
Crossref

8 words — < 1%

41 Naoya Kawakita, Hiroaki Toba, Keiko Miyoshi, Shinichi Sakamoto et al. "Bronchioalveolar stem cells derived from mouse-induced pluripotent stem cells promote airway epithelium regeneration", Stem Cell Research & Therapy, 2020
Crossref

8 words — < 1%

42 Shigeyuki Mukudai, Nao Hiwatashi, Renjie Bing, Michael Garabedian, Ryan C. Branski. " Phosphorylation of the glucocorticoid receptor alters signaling in vocal fold fibroblasts ", The Laryngoscope, 2018
Crossref

8 words — < 1%

43	academic.oup.com<br>Internet	8 words — < 1%
44	Advances in Geroscience, 2016.<br>Crossref	7 words — < 1%
45	Amisha V. Barochia, Maryann Kaler, Rosemarie A. Cuento, Elizabeth M. Gordon et al. " Serum Apolipoprotein A-I and Large High-Density Lipoprotein Particles Are Positively Correlated with FEV in Atopic Asthma ", American Journal of Respiratory and Critical Care Medicine, 2015<br>Crossref	7 words — < 1%
46	Ju Hyun Oh, Grace Hyun J. Kim, Jin Woo Song. "Interstitial lung abnormality evaluated by an automated quantification system: prevalence and progression rate", Respiratory Research, 2024<br>Crossref	7 words — < 1%
47	Stillwell, William. "Membrane Biogenesis", An Introduction to Biological Membranes, 2016.<br>Crossref	7 words — < 1%
48	"Sex-Based Differences in Lung Physiology", Springer Science and Business Media LLC, 2021<br>Crossref	6 words — < 1%
49	Adnan H. Gora, Saima Rehman, Jorge Dias, Jorge M. O. Fernandes, Pål A. Olsvik, Mette Sørensen, Viswanath Kiron. "Protective mechanisms of a microbial oil against hypercholesterolemia: evidence from a zebrafish model", Frontiers in Nutrition, 2023<br>Crossref	6 words — < 1%
50	Muhammed Fatih Kircali, Beste Turanli. "Idiopathic Pulmonary Fibrosis Molecular Substrates Revealed	6 words — < 1%

by Competing Endogenous RNA Regulatory Networks", OMICS: A Journal of Integrative Biology, 2023
Crossref

51	Paolo Comeglio, Annamaria Morelli, Luciano Adorini, Mario Maggi, Linda Vignozzi. "Beneficial effects of bile acid receptor agonists in pulmonary disease models", Expert Opinion on Investigational Drugs, 2017<br>Crossref	6 words — < 1%
52	Plant Biology and Biotechnology, 2015.<br>Crossref	6 words — < 1%
53	Weaver, Evelyn. "Metformin: A Potential Therapeutic to Correct Ovarian Dysfunction in the Broiler Breeder Hen", The Pennsylvania State University, 2023<br>ProQuest	6 words — < 1%
54	Willy Roque, Freddy Romero. "Cellular metabolomics of pulmonary fibrosis, from amino acids to lipids", American Journal of Physiology-Cell Physiology, 2021<br>Crossref	6 words — < 1%

EXCLUDE QUOTES	ON	EXCLUDE SOURCES	OFF
EXCLUDE BIBLIOGRAPHY	ON	EXCLUDE MATCHES	OFF
